# Different “metabolomic niches” of the highly diverse tree species of the French Guiana rainforests

**DOI:** 10.1038/s41598-020-63891-y

**Published:** 2020-04-24

**Authors:** Albert Gargallo-Garriga, Jordi Sardans, Victor Granda, Joan Llusià, Guille Peguero, Dolores Asensio, Romà Ogaya, Ifigenia Urbina, Leandro Van Langenhove, Lore T. Verryckt, Jérome Chave, Elodie A. Courtois, Clément Stahl, Oriol Grau, Karel Klem, Otmar Urban, Ivan A. Janssens, Josep Peñuelas

**Affiliations:** 1 CSIC, Global Ecology Unit CREAF-CEBAB-CSIC-UAB, Bellaterra, 08193 Catalonia Spain; 2 CREAF, Cerdanyola del vallès, 08193 Catalonia Spain; 3 Global Change Research Institute, Czech Academy of Sciences, Bělidla 986/4a, CZ-60300 Brno, Czech Republic; 4 University of Antwerp, Department of Biology, BE-2610 Wilrijk, Belgium; 5 Laboratoire Ecologie, évolution, interactions des systèmes amazoniens (LEEISA), Université de Guyane, CNRS, IFREMER, 97300 Cayenne, French Guiana; 6 INRA, UMR EcoFoG, CNRS, Cirad, AgroParisTech, Université des Antilles, Université de Guyane, 97310 Kourou, France

**Keywords:** Community ecology, Molecular ecology, Molecular biology

## Abstract

Tropical rainforests harbor a particularly high plant diversity. We hypothesize that potential causes underlying this high diversity should be linked to distinct overall functionality (defense and growth allocation, anti-stress mechanisms, reproduction) among the different sympatric taxa. In this study we tested the hypothesis of the existence of a metabolomic niche related to a species-specific differential use and allocation of metabolites. We tested this hypothesis by comparing leaf metabolomic profiles of 54 species in two rainforests of French Guiana. Species identity explained most of the variation in the metabolome, with a species-specific metabolomic profile across dry and wet seasons. In addition to this “homeostatic” species-specific metabolomic profile significantly linked to phylogenetic distances, also part of the variance (flexibility) of the metabolomic profile was explained by season within a single species. Our results support the hypothesis of the high diversity in tropical forest being related to a species-specific metabolomic niche and highlight ecometabolomics as a tool to identify this species functional diversity related and consistent with the ecological niche theory.

## Introduction

Tropical rainforests are characterized by amazingly high species diversity^1–3^. Numerous mechanisms have been proposed to explain the origin and the maintenance of such high tropical tree species coexistence on local scales^3,4^. Several factors such as soil heterogeneity^2,5^, micro-site singularities^6^, gradients of soil nutrient availability^2,7–9^, patchy distribution of the soil traits^10,11^, slope^12^, heterogeneity of disturbances and regeneration regimes^1^, topography^13^, or the long-term divergence of species-specific defences against herbivores^14^ have been identified as promoters of species coexistence and the maintenance of tree diversity in the tropics.

The concept of the Hutchinson multidimensional niche^15^ is a central theory used to explain species coexistence and shape communities composition, diversity and structure in ecological studies. The study and quantification of species niche at different spatial scales has been used to understand the functional diversity and ecological strategies of different species^16^. The classical way to estimate this functional niche is to focus on morphological and functional traits linked to the species strategy that are easier to measure in the field conditions (such as specific leaf area, foliar toughness, …). Numerous studies have combined this conceptual framework with functional foliar traits measurements to explain species coexistence, niche overlap level and thus the competition intensity^17–24^.

The metabolomic profile of a particular species results from the phenotypical expression of the species-specific combination of functional traits. Thus, by downscaling the “functional niche” theory to the molecular level, we here reduce the “niche” to the “metabolomic niche”. In a molecular approach each species should adjust its metabolome to ensure optimized biochemical and physiological functions at the homeostatic state given a certain amount of resources. In addition to this “homeostatic” species-specific metabolomic niche, each species should, moreover, have some degree of “flexibility” (phenotypic plasticity) to cope with environmental changing conditions such as with weather fluctuations (seasons) and climate shift. Each species, as a singular genotype which has been evolutionarily selected to live in a particular set of environmental abiotic and biotic circumstances^25^, should have a unique combination of levels of use of various elements^25^. Various metabolic pathways should thus operate at different levels in each species in order to optimize resource (nutrients, light, water) use efficiency, to provide fast and efficient acclimation of plants to changing conditions, and to ensure protection mechanisms against herbivores and diseases.

The metabolome of an organism is highly responsive to internal and external stimuli^26–29^ and is considered the final expression of an organism’s genotype and thus of organismic function at a particular moment^27,30,31^. Environmental metabolomics is one of the most recent “omic” technologies and is one of the most important recent developments in the study of plant ecophysiology^31,32^. It allows understanding how much variation in the metabolome is influenced by inherent genetic (constitutive) information and/or by external environmental factors^20,21,33–35^. Environmental metabolomics thus facilitates the understanding of plant processes, from phenotypic plasticity of each species population and thus adaptation capacity to shifts in community composition^36^. Each metabolite is involved in a metabolic pathway that consists of a series of biochemical reactions that are connected by their intermediates: the products of one reaction are the substrates for subsequent reactions, and so on. The shifts in the activities of the different metabolic pathways provide an overview of what is happening in a plant as a whole, for instance the up-regulation of metabolic pathways in secondary metabolism is related to an increased investment and/or allocation to defensive and/or anti-stress (drought, flooding, salinity, warming, frost, and so on) functions. In this way, a metabolomics profile provides a photography of the overall functional status of the organisms.

We hypothesized that in a complex highly diverse ecosystem where several potential competing species coexist such is the case of a wet tropical rainforest, a metabolome profile can be a useful tool to identify a species-specific functional niche and to “detect” and describe the potential functional niche shifts in changing environmental conditions with seasons. We hypothesized that each species has a unique species specific (genotype component) metabolomic profile corresponding to its position in the n-dimensional space (homeostatic component). But at the same time, the metabolomic niche should respond to some degree of flexibility (phenotypical component) to deal with the spatial and temporal variability of environmental conditions along spatial and temporal gradients. The up- and down-regulation of different metabolic pathways, from those of the primary metabolism including saccharides and amino acids to those of the secondary metabolism, including phenolics or terpenes, should provide a more solid understanding of the molecular responses of each species along spatial and seasonal variability (metabolomics niche flexibility). Accordingly, the screening of the plant metabolome should allow to prove the existence of a species’ “metabolomic niche”.

We used the analytical tools of environmental metabolomics to test the existence of this metabolomic niche for the species that coexist in the same habitats of French Guianese rainforests: We concretely aimed (i) to determine leaf metabolomic profiles of different tree species and (ii) to determine possible seasonal changes in these profiles.

## Results

We determined in field conditions the leaf metabolomic profiles of 54 French Guaiana species by analyzing 495 sampled individuals. The applied workflow allowed the determination of 1828 different metabolites (Data S1a, b; Supporting information) of which 876 could be identified (113 formally identified and 763 tentatively assigned) (Data S1a, b; Supporting information) and 952 remained unknown (defined as metabolites characterized by their mass spectra and their retention time). The identified metabolites were the most common metabolites whereas the tentative metabolites generally corresponded to rare compounds.

### “Metabolomic niche” of tree species

Species explained most part of the variance of the entire metabolomic data set (Table 1).

**Table 1:** PERMANOVA for the entire metabolomic data set for all species.

	Df	SumOfSqs	MeanSqs	F.Model	*R*^2^	Pr(>*F*)
Season	1	4.13	4.13	27.8	0.0879	0.001
Species	34	11.4	0.880	5.91	0.243	0.001
Season:Species	33	3.37	0.281	1.89	0.072	0.001
Residuals	137	20.4	0.149	0.434		
Total	187	47.0	1			

**Figure 1:**
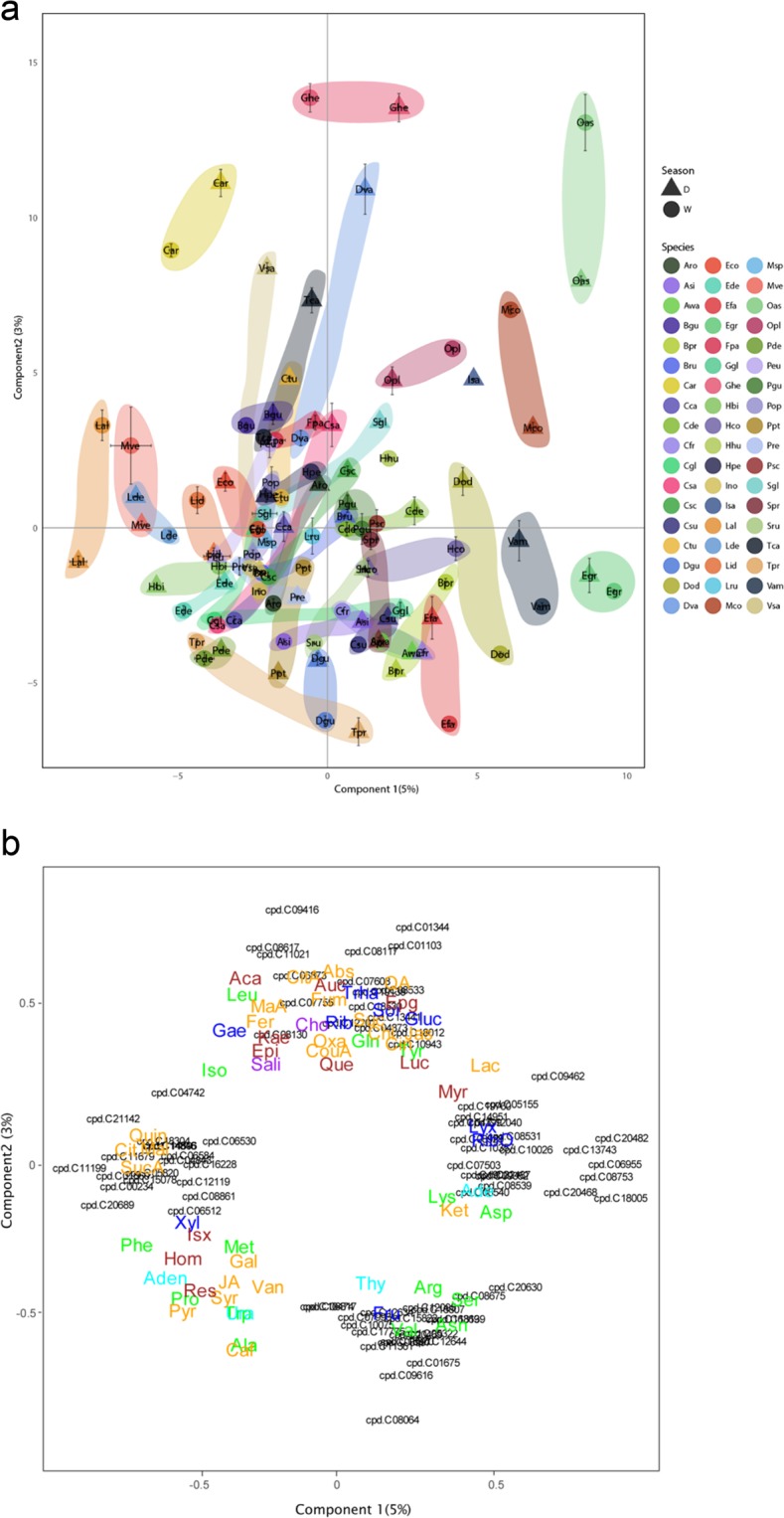
Partial least squares discriminant analysis (PLSDA) of the metabolomic data illustrating the samples (cases) belonging to different species (a) and variables (b; only the values higher than the 0.5 threshold are depicted). (a) Individual dots and each character indicate a different species, and the dots and triangles indicate dry and wet seasons respectively. The species are represented by centroids and the corresponding standard deviation: Oas, *Oxandra asbeckii*; Pde, *Protium decandrum*; Pop, *Protium opacum*; Cgl, *Caryocar glabrum*; Lal, *Licania alba*; Hbi, *Hirtella bicornis*; Cca, *Couepia caryophylloides*; Lde, *Licania densiflora*; Mco, *Moronobea coccinea*; Sgl, *Symphonia globulifera*; Tca, *Tapura capitulifera*; Csc, *Chaetocarpus schomburgkianus*; Vam, *Vouacapoua americaspe*; Hco, *Hymanea courbaril*; Efa, *Eperua falcata*; Egr, *Eperua grandiflora*; Dgu, *Dicorynia guianensis*; Pgu, *Paloue guianensis*; Ino, *Inga nouraguensis*; Dod, *Dipteryx odorata*; Bpr, *Bocoa prouacensis*; Bpr, *Alexa wachenheimii*; Ggl, *Goupia glabra*; Aro, *Aniba rosaeodora*; Sru, *Sextonia rubra*; Eco, *Eschweilera coriacea*; Ede, *Eschweilera decolorans*; Ghe, *Gustavia hexapetala*; Lid, *Lecythis idatimon*; Lpo, *Lecythis poiteaui*; Lza, *Lecythis zabucajo*; Hhu, *Hebepetalum humiriifolium*; Cfr, *Catostemma fragrans*; Spr, *Sterculia pruriens*; Lru, *Lueheopsis rugosa*; Csu, *Carapa surispemensis*; Bgu, *Brosimum guianense*; Hpe, *Helicostylis pedunculata*; Bru, *Brosimum rubescens*; Opl, *Osteophleum platyspermum*; Isa, *Iryanthera sagotiaspe*; Msp, *Myrcia splendens*; Asi, *Agonandra silvatica*; Psc, *Pogonophora schomburgkiaspe*; Dva, *Drypetes variabilis*; Cde, *Capirona decorticans*; Fpa, *Ferdinandusa paraensis*; Ctu, *Chimarrhis turbispeta*; Tpr, *Talisia praealta*; Car, *Chrysophyllum argenteum*; Peu, *Pouteria eugeniifolia*; Ppt, *Pradosia ptychandra*; Pre, *Pouteria retinervis*; Csa, *Chrysophyllum sanguinolentum*; Mve, *Micropholis venulosa* and Vsa, *Vochysia sabatieri*. (b) Loadings of the metabolites; only those with loadings>0.5 are depicted. The various metabolomic families are represented by colors: dark blue, sugars; green, amino acids; orange, compounds involved in the metabolism of amino acids and sugars; cyan, nucleotides; brown, phenolics and red, others. Metabolites: arginine (Arg), asparagine (Asn), aspartic acid (Asp), glutamic acid (Glu), glutamine (Gln), isoleucine (Ile), lysine (Lys), leucine (Leu), methionine (Met), phenylalanine (Phe), serine (Ser), tryptophan (Trp), threonine (Thr), tyrosine (Tyr), valine (Val), adenine (Ade), adenosine (Ado), thymidine (TdR), chlorogenic acid (CGA), trans-caffeic acid (CafA), α-ketoglutaric acid (KG), citric acid (Cit), L-malic acid (Mal), lactic acid (Lac), abscisic acid (Abs), pyruvate (Pyr), succinic acid (SAD), pantothenic acid hemicalcium salt (Pan), jasmonic acid (JA), 5,7-dihydroxy-3,4,5–trimethoxyflavone (Fla), acacetin (AC), epicatechin (EC), epigallocatechin (EGC), homoorientin (Hom), isovitexin (Ivx), kaempferol (Kae), myricetin (Myr), quercetin (Qct), resveratrol (Rvt), saponarin (Sp), catechin hydrate (Cat), 3-coumaric acid (CouA), gallic acid (GA), quinic acid (QuiA), sodium salicylate (Sal), syringic acid (Syr), trans-ferulic acid (Fer), vanillic acid (Van), 2-deoxy-D-ribose (Rib), D-(-)-lyxose (Lyx), D-( + )-sorbose (Sor), D-( + )-trehalose dehydrate (Tre) and aucubin (Auc). The other metabolites with the prefix “cpd” were tentatively identified using the KEGG database (Table S1). The names of these compounds are in the Supporting information and in the KEGG webpage (https://www.genome.jp/kegg/pathway.html).

A PLSDA of all detected metabolites (Fig. 1a; Data S1a, Supporting information) separated the species and the seasons sampled. The PLSDA discriminated the species along the two first axes (Component 1 and Component 2), whereas the seasons were separated mainly along Component 2. Each species tended to maintain its own species-specific position in the biplot, despite seasonal shifts in several species. Species differed in the magnitude of their seasonal shift. The figure shows that each species has a distinct “homeostatic species-specific metabolomic profile” and a flexible (rather idiosyncratic) species metabolomic response to seasonality, thus confirming the “metabolomic niche” hypothesis

The species with the most positive scores along the Component 1 axis were *Oxandra asbeckii*, *Vouacapoua americaspe*, *Moronobea coccinea* and *Eperua grandiflora*. The metabolic pathways with the most positive scores in Component 1 were those of the biosynthesis of pantothenate and CoA, flavone and flavonol, and anthocyanin. The species with the most negative scores on the Component 1 were *Licania alba*, *Micropholis venulosa* and *Licania densiflora*. The metabolic pathways with the most negative scores in the Component 1 were those of amino sugars and nucleotide sugars metabolism and Isoquinoline alkaloid biosynthesis.

The species with the highest positive scores along the Component 2 axis were *Gustavia hexapetala* and *Chrysophyllum argenteum*, and the metabolic pathways associated with positive scores along Component 2 were toluene degradation, biosynthesis of type II polyketide products, chloroalkane and chloroalkene degradation, isoflavonoid biosynthesis, ethylbenzene degradation, one carbon pool by folate and dioxin degradation. The species with negative values along the Component 2 axis were *Dicorynia guianensis*, *Eperua falcata* and *Talisia praealta*, and the metabolic pathways that loaded strongly toward negative values were thus purine metabolism; pyrimidine metabolism; carotenoid biosynthesis; alanine, aspartate and glutamate metabolism; terpenoid backbone biosynthesis; tropane, piperidine and pyridine alkaloid biosynthesis; acarbose and validamycin biosynthesis and novobiocin biosynthesis.

**Figure 2:**
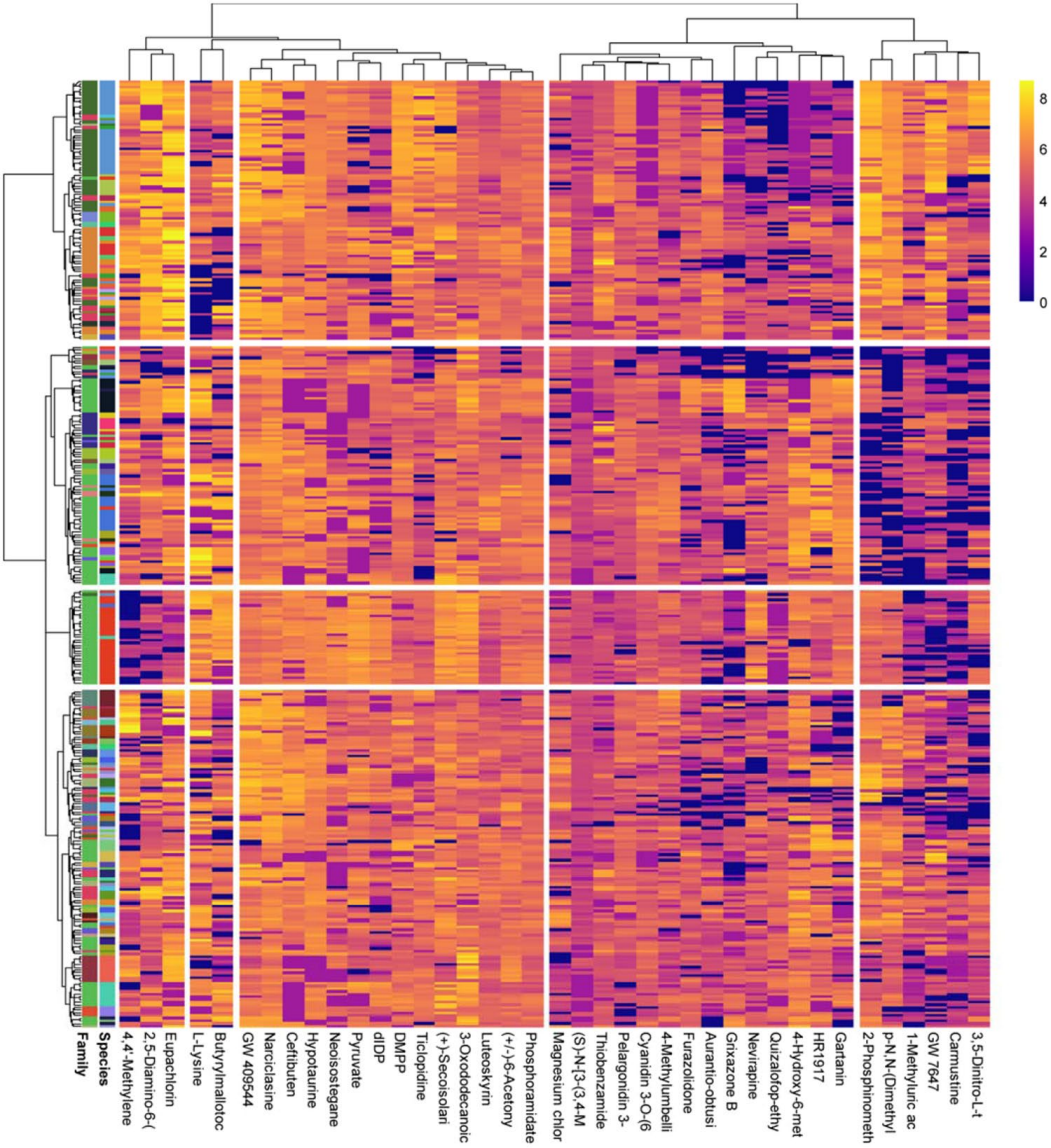
Heatmap-clustering analysis of the metabolites identified in the foliar metabolomes of families and species. The metabolites were identified/assigned based on the KEGG database or our library. Family and each species generally clustered together with the corresponding metabolites. The samples are represented horizontally, and the metabolites are represented vertically on the heatmap. Abundance is represented by the intensity of the color of each metabolite, with blue representing low abundance and yellow representing high abundance. The 50 most important metabolites that were differentially affected (*P* < 0.05) between species and family are represented.

The differences among species discriminated by PLSDA were very large and masked the smaller intraspecific differences (Fig. 1). Species were significantly separated by a heatmap-clustering analysis of all species (Fig. 2). The heatmap clustered the 50 most species-discriminant metabolites in the regression tree analysis.

### Season

**Figure 3:**
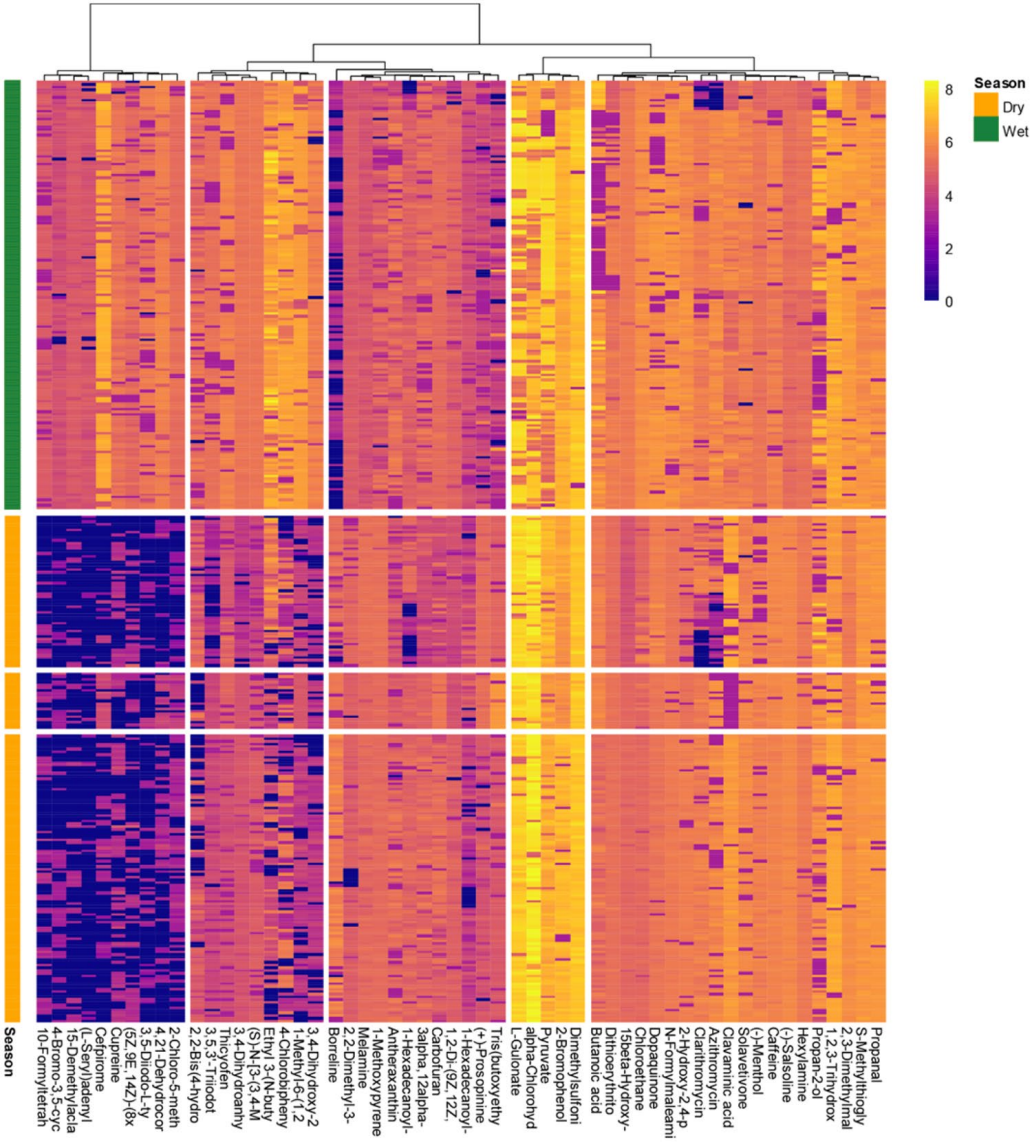
Heatmap-clustering analysis of the 50 most important metabolites identified by the Random Forest analysis that were best explained by season. The factors (Dry and Wet) are represented by different colors (left).

The heatmap-clustering analyses using the results of the regression tree analysis showed the differential importance of season in explaining the variance of the metabolome (Fig. 3).

The complexity of the metabolomic data from the various species and seasons can be reduced by determining the differences between and among the metabolic pathways (Table S2, Supporting information). We could thus identify the metabolic pathways that are overexpressed and inhibited for each level of each independent variable. Our identified metabolites belonged to a total of 47 pathways in the KEGG database.

The metabolic pathways associated with lysine, tyrosine and isoflavone biosynthesis, energy metabolism and xenobiotic biodegradation were upregulated in the wet season relative to the dry season. In contrast, the pathway for lipid metabolism was more upregulated in the dry than the wet season(Table S2).

## Discussion

Combining LC-Orbitrap-MS, bioinformatics tools, and data mining was highly efficient and enabled us to determine that tree taxonomic family and species and season had different metabolomic profiles in consonance with the “metabolomics niche” hypothesis.

Species niches are characterized by different quantities of basic abiotic resources (light, water and nutrients) and by biotic relationships. Plant species consequently subdivide space along gradients of quantitative variations in light, water and nutrients. These variations are often associated with variations in altitude, slope, aspect, soil type and/or interaction with other plant species. Previous studies have identified associations between sets of species and particular habitat conditions in tropical rainforests^37–40^, e.g. broad variations in soil properties (such as parental material, drainage and mounds and hollows at the roots of fallen trees), soil nutrient availability^2^ and topography (such as ridges, steep slopes and creek margins), which is consistent with Hutchison´s approach of niche theory^41^.

Identifying variables that provide a global view of niche partitioning between different species in the same ecosystem communities, however, has been difficult. The proposed biogeochemical niche hypothesis^42–44^ claims that each species has an optimal elemental composition and stoichiometry linked to its optimal function in its specific ecological biogeochemical niche^25^. This optimal elemental composition is due to the differences in functions and morphologies developed over a long period, so each species tends to reach an optimal chemical composition linked to a singular optimal function (homeostasis). Plant species should also be, to some degree, flexibly adaptable to alter their elemental stoichiometries in response to changes in the composition of neighboring species and/or in environmental conditions (such as climatic gradients)^45–47^, probably with a tradeoff between adaptive capacity (flexibility) and stability (homeostasis)^48^.

This study thus provides consistent evidence of a new way to characterize niche according to plant metabolome. The “metabolomic niche” would be due to niche differentiation at the level of the species-specific functional adaptation to specific positions along natural gradients of biotic and abiotic factors. Tropical trees may have finely subdivided the niche^49^, but little direct evidence currently supports a measurable variable of the overall functional differences among sympatric species.

Our results clearly indicate that species specificity could explain most of the variance of a metabolomic profile, despite the environmental differences involved in this study (season, site micro-conditions, and topography). A previous study in a Panamanian rainforest also reported differences in metabolomic profiles of sympatric dominant tree species^20^. However, to the best of our knowledge, our study analyses for the first time a broad set of tropical-forest tree species growing in different sites, topographic conditions, and across seasons. Such an extensive data set allowed us to prove the existence of species metabolome identity. Functional metabolomic niches could thus be detected using metabolomic analyses. They could also help us to understand the mechanisms and environmental circumstances underlying the high niche speciation in tropical forests by comparing the up- and down-regulation of metabolic pathways among species. The intraspecific variation has not been tested in this study, but it has been observed in the multivariate analyses that different individuals of the same species have similar but no exact overall foliar elemental composition.

Our results also identified significant additional metabolomic differences due to species flexibility to acclimate to the strong environmental variability associated with wet and dry seasons. The number and type of metabolites in the tree species studied were tightly associated with fluctuating seasonal activities. For example, metabolic pathways involved in cell production were substantially up-regulated in wet as compared to dry season. We have also found that trees up-regulate different secondary metabolic pathways during wet and dry seasons (Fig. 3). These results are consistent with previous studies, where primary metabolism was generally up-regulated in seasons favourable for plant productivity or active growth^29,33^. The two groups of seasonal factor defined by our metabolomic profiling were also clearly separated in the cluster mappings.

The high amounts of some secondary metabolites in the dry season, such as phenolics, are consistent with a response of trees to drought stress^50^. Particularly flavonoids received a great attention due to their antioxidant capacity^51,52^. These compounds are involved in many protective physiological mechanisms against oxidative stress (drought, excessive light intensity)^53^, and/or control interactions of plants with microbes, animals or other plants^54,55^. Our results show that flavonoids accumulate during the dry season (Fig. 1), as previously reported^56–59^. Such accumulation of flavonoids might therefore reflect an increased demand for plant protection against oxidative stress associated with substantially higher daily maxima and total sums of solar radiation during dry than wet season^60–62^.

The amounts of other secondary compounds, such as anthocyanins malvidin, peonidin glucoside and taxifolin (quercetin), were higher during wet than dry season (Fig. 3). This increase may be due to the antioxidant function of anthocyanin and taxifolin and the need for an additional protection against hypoxic conditions following frequent flooding. This possibility implies either that plants synthesize these phenolic compounds in response to hypoxic stress, phenolics are readily available due to increased detrital leaching during the wet season, and are absorbed by plant roots^63^. Hypoxic conditions may not be stressful to adapted plants but may facilitate recycling of protective phenolic compounds^63^. Nevertheless, the amounts of several phenolic compounds have been shown to increase in plants exposed to severe anoxic conditions^63–65^. For example, the amount of plant hormone sorgolactone (structural derivative of sesquiterpenes) modulating the formation of stems, leaves, and flowers as well as the development and ripening of fruits^66,67^ is substantially increased in wet as compared to dry season.

## Conclusions and final remarks

This study found very significant species-specific differences in metabolic profiles in a broad set of sympatric tree species in French Guiana. This result is consistent with the niche theory in this highly diverse tropical ecosystem and also demonstrates the usefulness of metabolomics to analyse the potential differences in the functions of plant species in a community to detect different functional niches. We suggest that species occupy “metabolomic niches”. This “metabolomic niches” would be associated with an optimal function in an environmental niche (resulting from a set of light conditions, water and nutrients availability, and/or specific biotic relationships) acquired after a long-term uninterrupted evolutionary process in this old tropical ecosystem. The differential use of basic abiotic factors and distinct species-specific biotic relationships should allow the coexistence of species by reducing direct competition and maximize the overall use of resources by the community. Moreover, our results also show that seasonal environmental changes can shift the species-specific metabomic profile but still maintaining the species metabolomic niche in the n-dimensional metabolomics space. This study, thus, despite its spatial (only two tropical forests) and organic (only foliar analyses) limitations, provides significant evidences of the suitability of the use of metabolomics tools as a new way to advance in a wide array of ecological studies providing a measurable method to identify genotype populations niche and following its dynamics and response/adaptation mechanisms in changing environments.

## Materials and methods

### Study area

French Guiana lies between 2°10′ and 5°45′N and 51°40′ and 54°30′W. Ninety-seven percent of the region is covered by lowland wet tropical forest (Chave *et al*., 2001). A pronounced dry season extends from September to November, associated with the displacement of the inter-tropical convergence zone. The mean annual temperature is 25.8 °C, with an annual amplitude of 2 °C and a daily amplitude of 7 °C in the rainy season (10 °C in the dry season)^68,69^. Field work was conducted in two old-growth rainforests, at the Paracou Research Station (5°18′N, 52°53′W) and the Nouragues Research Station (4°05′N, 52°40′W). Mean annual rainfall is 2990 mm at Nouragues and 3160 mm at Paracou (Aguillos *et al*. 2018). For this study, we picked three topographical positions in each site: (1) top of hills (top hill), (2) the middle of the slopes at intermediate elevation (middle slope) and (3) bottom end of the slopes, at low elevation, just above the creek (bottom slope) (Table 1).

### Study plots

At each site, four plots of 20 × 20 m per topographical position were established (distances between plots of 10–200 m) in the vicinity of long-term undisturbed plots that have been monitored at both sites for 30 years (Fig. 1). The sand content was higher, and the clay content was lower in the bottom slope plots than in the top hill and middle slope plots at both sites^69^.

### Sample collection

Leaves were sampled once per season at both Paracou and Nouragues: the same individuals were sampled in June (wet season) and September (dry season) in 2015. Two to six individuals of the three dominant tree and two smallest species were chosen for each site, season, topography (we distinguished among Top hill, Middle slope, Bottom slope) and plot (we had four plots for each one of these topographies and sites), depending on the individuals available (2 sites × 2 seasons × 3 topographies × 4 plots × 2–6 trees). We collected leaves always in the same position and always of similar age (mature middle-age leaves) 495 trees from 54 species (Tables S3) with the help of professional climbers. Large numbers of species in metabolomic studies require a rapid technique and appropriate storage for subsequent analyses^70–73^. We sampled about 2 g of leaf tissues from each tree. Leaves were put immediately into a paper can, frozen in liquid nitrogen, and transported to the laboratory.

### Leaf processing for metabolomic analysis

The leaves were processed as described in detail by Rivas-Ubach *et al*. (2013). Briefly, the leaves frozen in liquid nitrogen were lyophilized and stored in paper cans at −80 °C. The samples were ground with a ball mill at 1500 rpm for 3 min, producing a fine powder that was then stored at −80 °C until extraction of the metabolites.

### Liquid chromatography Orbitrap mass spectrometry analysis

The homogenized samples were then extracted using a methanol: H_2_O solution (1:1). The extracted fraction of each sample was analyzed twice by LC-Orbitrap-MS, first using the positive ion mode and then the negative ion mode. Recordings from both the diode array detection (DAD) detector and the high-resolution mass spectrometer were monitored and saved to verify system function and to evaluate subsequent results.

A Dionex UltiMate 3000 chromatographic system (Thermo Fisher Scientific, MA, USA) was used for high-performance liquid chromatography. The LC-Orbitrap-MS system (controlled by Xcalibur version 2.2, Thermo Fisher Scientific Corporation) was run in gradient mode using a 150 ×2.1 mm 3 µm Hypersil Gold reverse-phase column (Thermo Fisher Scientific), set at 30 °C. Solvent A was acetonitrile and solvent B was 0.1% acetic acid. Both A and B mobile phases were filtered and degassed for 10 min in an ultrasonic bath prior to use. Gradient elution chromatography was performed starting with 10% A and 90% B and held for 5 min. The percentage of A was increased to 90% within 5–20 min. This composition was then maintained for 5 min, after which the system was equilibrated to initial conditions (10% A and 90% B) over 5 min. A flow rate of 0.3 mL/min was used. MS and MS/MS were performed using an LTQ Orbitrap XL high-resolution mass spectrometer (ThermoFisher Scientific) equipped with a HESI II (heated electrospray ionization) source. The high-resolution mass spectrometer was operated in full-scan mode with a resolution of 60 000. Full-scan spectra were acquired for the mass range m/z 50–1000 in the positive mode and 65–1000 in the negative mode. The resolution and sensitivity of the Orbitrap were controlled by the injection of a mixed standard after the analysis of each batch (30 samples), and the resolution was also checked with the aid of lock masses (phthalates). Blanks were also analyzed during the sequence. Samples were reanalyzed (MS/MS) under the same conditions, but with selecting the top three parent ions of each scan. Parent ions with a minimum peak area of 500 were fragmented by CID (collision-induced dissociation) using a normalized collision energy of 35, activation Q 0.25 and activation time 90 ms and analyzed in ITMS.

### Metabolite identification

Metabolites that were defined after the comparison with our standard compound library or by a matching of MS/MS data were considered to be reliably identified. The metabolites were identified against our library (>200 compounds) and were further confirmed by mass, retention time, isotopic pattern and ring double-bound parameters (Table S1). A second approach identified the individual peaks using the KEGG^74^ database with a built-in M/Z MINE utility with an m/z threshold of 5 ppm. A third approach used MASSBANK^75^ databases, searching for each MS and MS/MS. We tentatively assigned metabolites to molecular ions with exact masses corresponding to metabolites identified in the databases. Remaining unidentified metabolites were characterized “metabolites” characterized by their mass spectra and their retention time. Table S1 (Supporting information) includes the detailed interpretation of the experimental MS/MS spectra that supported our tentative identifications of the masses referred to throughout the manuscript that were not positively identified.

### Chromatogram alignment and metabolite quantification

The raw data from the LC-Orbitrap-MS were processed and compared using XCMS^76^. Data in an instrument-specific format (.raw) were converted to a common data format (.mzXML). XCMS was used for the nonlinear alignment of the data in the time domain and the automatic integration and extraction of the peak intensities. The **peak detection** algorithm we used was based on cutting the LC/MS data into slices a fraction of a mass unit (0.1 m/z) wide and then operating on the individual slices in the chromatographic time domain. **Peak Matching**. Peaks were identified in the individual samples and then matched across samples to calculate the deviations of retention time and to compare relative ion intensities. Peaks were smoothed and deconvoluted using a local minimum search algorithm (95% chromatographic threshold, minimum retention range of 0.2 min, minimum relative height of 5% and minimum ratio top/edge of 0.5). Chromatograms were aligned using the algorithm with a tolerance of 5 ppm of m/z and 0.2 min of retention time. Normalized peak areas were used for quantification, and their values were log-transformed before statistical analysis (Supporting information). MS/MS data were only used for metabolite identification.

### Statistical and bioinformatic analysis

The metabolites were quantified after eliminating peaks that were not consistently representative (mass/RT present in at least three samples of any provenance; each sample was split and randomly queued into two LC-MS run batches). The peak areas corresponding to each metabolite were normalized based on total peak areas in the sample. Neutral masses obtained in positive and negative modes were evaluated to avoid duplicates (same retention time and neutral mass in the different modes), retaining the most intense peaks.

To test the “metabolomic niche hypothesis” with its homeostatic and flexible (seasonal) components, we used ADONIS which implements a multivariate non-parametric ANOVA of the LC-MS data using Euclidean distances, with species and season (wet and dry) as fixed factors. Previous exploratory ADONIS multivariate analysis showed that neither site nor topography had significant differentiating effects on the metabolomes. The number of permutations was set at 2000. We characterized and visualized the differences among species and between seasons and the metabolites most responsible for these differences by scaling normalized areas of different metabolite peaks and subjected them to principal component analysis (PCA), sparse partial least squares(sPLS), partial least squares discriminant analysis (PLSDA) and heatmap-clustering analyses for processing the “omic” data sets^76^. A regression tree analysis (Random Forest) was also performed with 2000 trees to identify the metabolites that are best explained by the factors included in the analysis.

All statistical procedures were performed using R v3.5.1. Core software using the SEQKNN, DOBY, PHEATMAP, VEGAN, FACTOEXTRA, FACTOMINER, DPLYR, RANDOMFOREST and MIXOMICS packages.

## Supplementary information

Supporting information.
