# Supplementary material for: Different “metabolomic niches” of the highly diverse tree species of the French Guiana rainforests
Source: Sci Rep. 2020 Apr 24;10:6937. doi: 10.1038/s41598-020-63891-y (PMC7181821; doi:10.1038/s41598-020-63891-y)
Supplement: Supplementary file 1 — Supporting information. [file 41598_2020_63891_MOESM1_ESM.pdf]

Different “metabolomic niches” of the highly diverse tree species of the French Guiana rainforests

Albert Gargallo-Garriga[1,2,3*], Jordi Sardans[1,2], Victor Granda[1,2], Joan Llusià[2,3], Guille Peguero[1,2,4], Dolores Asensio[2,3], Romà Ogaya[2,3], Ifigenia Urbina[2,3], Leandro Van Langenhove[4], Lore T. Verryckt[4], Jérome Chave, Elodie A. Courtois[4,5], Clément Stahl[6], Oriol Grau[1,2], Karel Klem[3], Otmar Urban[3], Ivan A. Janssens[4], Josep Peñuelas[1,2].
[1]CSIC, Global Ecology Unit CREAF-CEBAB-CSIC-UAB, Bellaterra 08193, Catalonia, Spain. [2]CREAF, Cerdanyola del vallès 08193, Catalonia, Spain. [3]Global Change Research Institute, Czech Academy of Sciences, Bělidla 986/4a, CZ-60300 Brno, Czech Republic.[4]University of Antwerp, Department of Biology, BE-2610 Wilrijk, Belgium, [5] Laboratoire Ecologie, évolution, interactions des systèmes amazoniens (LEEISA), Université de Guyane, CNRS, IFREMER, 97300 Cayenne, French Guiana, [6] INRA, UMR EcoFoG, CNRS, Cirad, AgroParisTech, Université des Antilles, Université de Guyane, 97310 Kourou, France.

Table S1. Metabolites that were defined after the comparison with our standard compound library or by a matching of MS/MS data were considered to be reliably identified using the KEGG and MASSBANK platforms.

Detection	MS	RT	Comp_code	Comp_name
Int. Sta.	106.05	1.46	[M+H]+	Arginine
Int. Sta.	90.05	1.47	[M+H]+	Asparagine
Int. Sta.	175.12	1.47	[M+H]+	Aspartic acid
Int. Sta.	116.07	1.43	[M+H]+	Glutamic acid
Int. Sta.	118.09	1.34	[M+H]+	Glutamine
Int. Sta.	132.1	1.49	[M+H]+	Glycine
Int. Sta.	132.1	1.53	[M+H]+	Histidine
Int. Sta.	147.08	1.7	[M+H]+	Isoleucine
Int. Sta.	150.06	1.76	[M+H]+	Leucine
Int. Sta.	147.11	1.46	[M+H]+	Lysine
Int. Sta.	166.09	1.59	[M+H]+	Methionine
Int. Sta.	182.08	1.32	[M+H]+	Phenilalanine
Int. Sta.	205.1	1.91	[M+H]+	Proline
Int. Sta.	117.02	1.54 a 1.77	[M+H]+	Serine
Int. Sta.	193.05	2.49	[M+H]+	Threonine
Int. Sta.	193.03	1,74, 1,78	[M-H]-	Tryptofan
Int. Sta.	163.04	10.57	[M-H]-	Tyrosine
Int. Sta.	193.07	1.75	[M+H]+	Valine
Int. Sta.	87.05	11.29	[M-H]-	Adenine
Int. Sta.	187.04	1.51	[M+H]+	Adenosine
Int. Sta.	155.14	7.71	[M-H]-	Guanine
Int. Sta.	169.04	11.29	[M+H]+	α-Ketoglutaric acid
Int. Sta.	171.02	16.63	[M+H]+	Chlorogenic acid(-)
Int. Sta.	117.07	4.65	[M+H]+	Citric acid (anhydrous)
Int. Sta.	115.06	1,55 a 1,83	[M+H]+	L-(-)-Malic acid
Int. Sta.	106.05	8.96	[M+H]+	Lactic acid
Int. Sta.	168.07	9.63	[M+H]+	Pyruvic acid
Int. Sta.	149.06	1.47	[M+H]+	Succinic acid
Int. Sta.	181.06	1,42 and 1,64	[M-H]-	Tartaric acid
Int. Sta.	193.03	14.28	[M+H]+	Abscisic acid (ABA)
Int. Sta.	193.07	4.82	[M+H]+	Ascorbic acid
Int. Sta.	217.05	1.75	[M+H]+	Pantothenic acid hemicalcium salt
Int. Sta.	199.06	1.51	[M+H]+	(+)-Catechin hydrate (anhydrous)
Int. Sta.	282.08	10.57	[M+H]+	3-coumaric acid
Int. Sta.	211.13	4.9	[M+H]+	5,7-dihydroxy-3,4,5-trimethoxyflavone
Int. Sta.	355.08	1,46 and 1,77	[M-H]-	Acacetin
Int. Sta.	265.14	14.81	[M+H]+	D-Pinitol
Int. Sta.	149.05	3,11	[M+H]+	Epicatechin
Int. Sta.	305.06	13.57	[M+H]+	Epigallocatechin
Int. Sta.	113.03	1.44	[M-H]-	Gallic acid
Int. Sta.	136.06	11,09	[M+H]+	Homoorientin
Int. Sta.	243.1	1.5	[M+H]+	Isovitexin
Int. Sta.	268.1	1,42 and 1,77	[M+H]+	Kaempferol

Int. Sta.	149.05	1,48 and 1,81	[M+H]+	Luteolin
Int. Sta.	149.05	1,49 and 1,75	[M+H]+	Myricetin
Int. Sta.	191.02	1.43	[M-H]-	Protocatechuic acid
Int. Sta.	149.05	1.44	[M-H]-	Quercetin
Int. Sta.	149.05	1.77	[M-H]-	Quinic acid
Int. Sta.	87.01	1.42	[M-H]-	Sinapic acid
Int. Sta.	179.06	1.43	[M-H]-	Sodium salicylate
Int. Sta.	87.01	1.65	[M-H]-	Syringic acid
Int. Sta.	353.09	1.44	[M-H]-	trans-Ferulic acid
Int. Sta.	229.09	1.65	[M-H]-	Vanillic acid
Int. Sta.	285.08	3.11	[M-H]-	D-(-)-Lyxose
Int. Sta.	287.06	13.09	[M+H]+	D-(+)- Raffinose pentahydrate
Int. Sta.	291.09	16.87	[M+H]+	D-(+)-Sorbose
Int. Sta.	303.05	14.82	[M+H]+	D-(+)-Trehalose dihydrate
Int. Sta.	307.08	4,93 a 5,20	[M+H]+	Humulene
Int. Sta.	319.04	13.72	[M+H]+	Terpinene
Int. Sta.	151.06	1,54 a 2,64	[M+H]+	Terpineol
Int. Sta.	433.11	12.47	[M+H]+	Caryophyllene oxide
Int. Sta.	449.11	1.45	[M-H]-	Linalool
KEGG	132.1	860.98	cpd:C21184	(-)-5'-Demethylyatein
KEGG	272.25	1456.42	cpd:C11878	(-)-Abietadiene
KEGG	264.14	698	cpd:C11060	(-)-Abscisic acid
KEGG	328	1061.01	cpd:C10198	(-)-Acanthocarpan
KEGG	383	79.69	cpd:C09462	(-)-alpha-Hydrastine
KEGG	264.2	1332.51	cpd:C09036	(-)-Apparicine
KEGG	159.09	94.63	cpd:C08269	(-)-Betonicine
KEGG	330.1	88.19	cpd:C03040	(-)-Bisdechlorogeodin
KEGG	267.1	108.64	cpd:C09368	(-)-Caaverine
KEGG	360.16	820.68	cpd:C20454	(-)-Lariciresinol
KEGG	432	1721.51	cpd:C16223	(-)-Medicocarpin
KEGG	156	1760.37	cpd:C00400	(-)-Menthol
KEGG	286.08	87.15	cpd:C10508	(-)-Nissolin
KEGG	317.28	1101.62	cpd:C10777	(-)-Ormosanine
KEGG	358.1	100.8	cpd:C20455	(-)-Pinoresinol
KEGG	282.2	1144.39	cpd:C09235	(-)-Quebrachamine
KEGG	193.11	1724.65	cpd:C09640	(-)-Salsoline
KEGG	286.12	1000.68	cpd:C10526	(-)-Sativan
KEGG	300	1793.84	cpd:C16228	(-)-Sophorol
KEGG	326	1061.49	cpd:C12260	(-)-Tortuosamine
KEGG	393.2	701.31	cpd:C10620	(-)-Tylocrebrine
KEGG	344.1	144.44	cpd:C10101	(-)-Usnic acid
KEGG	400.15	645.42	cpd:C17845	(+)-Aschantin
KEGG	594.27	1129.79	cpd:C11141	(+)-Atherospermoline
KEGG	325.13	109.85	cpd:C09389	(+)-Cassythicine
KEGG	290	98.42	cpd:C06562	(+)-Catechin
KEGG	288.06	747.37	cpd:C10415	(+-)-Dalbergioidin
KEGG	370.1	633.32	cpd:C17844	(+)-Demethoxyaschantin
KEGG	386.17	1029.07	cpd:C10561	(+)-Eudesmin
KEGG	340.13	813.65	cpd:C10616	(+)-Galbacin
KEGG	462	73.88	cpd:C12254	(+)-Plicamine

KEGG	313.26	1199.79	cpd:C10167	(+)-Prosopinine
KEGG	362.2	1091.82	cpd:C20456	(+)-Secoisolariciresinol
KEGG	418.16	651.11	cpd:C10889	(+)-Syringaresinol
KEGG	580.2	668.55	cpd:C10890	(+)-Syringaresinol O-beta-D-glucoside
KEGG	389.13	443.76	cpd:C12201	(+/-)-6-Acetonyldihydrosanguinarine
KEGG	350	1721.5	cpd:C17496	(10)-Gingerol 8 11-cis-13-trans-eicosatetraenoate
KEGG	284.2	1056.19	cpd:C16505	(10S)-Juvenile l 2-dihydronaphthalene
KEGG	220.18	1287.67	cpd:C20325	(11R)-Dihydroa 5'R)-5'-Hydroxyaverantin
KEGG	320	988.49	cpd:C04742	(15S)-15-Hydrc 3-Dihydroxybenzoyl)adenylate
KEGG	451.14	673.99	cpd:C14793	(1R)-Glutathior 24(1)R)-Fucosterol epoxide
KEGG	388.12	219.41	cpd:C20501	(1'S6E)-Farnesol
KEGG	483.1	620.72	cpd:C04030	(2,3-Dihydroxybenzoyl)adenylate
KEGG	428.37	1661.75	cpd:C03910	(24R4-benzoxazin-3(4H)-one
KEGG	222	245.02	cpd:C01126	(2E 2'S)-Oscillol
KEGG	280.1	118.35	cpd:C15191	(2-Isopropyl-1- 3S)-2-Hydroxyt 2 3-tricarboxylate
KEGG	373.1	406.59	cpd:C15768	(2R)-2-beta-D-(6Z)-Farnesyl diphosphate
KEGG	600.42	1382.43	cpd:C16274	(2S 2'S)-Myxol 2'-(: 4-di-O-methyl-alpha-L-fucoside)
KEGG	332.18	718.44	cpd:C04655	(2S 9E 14Z)-(8xi 11xi 12-Trihydroxyicosa-5
KEGG	382	79.96	cpd:C19760	(2Z 4 6-trihydroxy-5- 4-cyclohexadien-1-one
KEGG	758.5	1404.23	cpd:C15937	(3R 6-tetrahydronicotinamide-adenine dinucleotide
KEGG	354.2	1351.75	cpd:C04843	(5Z 7S)-6 7-Epoxyoctadecanoic acid
KEGG	346.1	689.96	cpd:C15591	(S)-usnate
KEGG	683	77.28	cpd:C20482	(R)-NADH-hydrate
KEGG	298.25	1242.32	cpd:C13791	(6R 13-Epoxyoctad 11-dienoic acid
KEGG	386.08	96.09	cpd:C15666	(7R)-7-(4-Carbo 12Z)-(8R)-Hydr 12-dienoic acid
KEGG	310.2	1043.27	cpd:C20704	Linoleate
KEGG	294.22	1240.85	cpd:C04594	(9Z)-(13S)-12,13-Epoxyoctadeca-9,11-dienoate
KEGG	296.24	1204.56	cpd:C08318	Laetisaric acid
KEGG	301	79.06	cpd:C02487	(Ac)2-L-Lys-D-Ala
KEGG	255.09	95.24	cpd:C14622	(E)-1-Methoxy-4-[2-(4-nitrophenyl)ethenyl]benzene
KEGG	434	1720.65	cpd:C05820	(L-Seryl)adenylate
KEGG	349.1	93.87	cpd:C11749	(p-Aminobenzyl)penicillin
KEGG	85.09	107.25	cpd:C20809	(R)-2-Methylpyrrolidine
KEGG	217.1	105.7	cpd:C21289	(R)-3-(indol-3-yl)-2-oxobutyrate
KEGG	402.09	93.62	cpd:C04352	(R)-4'-Phosphopantothenoyl-L-cysteine
KEGG	88.1	108.78	cpd:C00810	(R)-Acetoin
KEGG	339.1	88.57	cpd:C11818	(R)-Canadine 3-Epoxysqualene
KEGG	266.13	110.76	cpd:C14546	(R)-Indenestrol 5-dihydrofuran-2-acetate
KEGG	90	1790.53	cpd:C00256	(R)-Lactate
KEGG	426.39	1509.05	cpd:C01054	lupan-3beta
KEGG	142.03	5.32	cpd:C14610	(S)-5-Oxo-2 4-Methylenedioxyphenyl)-2-(mercaptomethyl)-1-oxoprolyl]glycine
KEGG	158.03	78.86	cpd:C00337	(S)-Dihydroorotate
KEGG	486	1743.92	cpd:C01315	(S)-4-Methylenedioxyphenyl)-2-(acetylthio)methyl-1-oxoprolyl]glycine benzyl ester
KEGG	354.1	208.98	cpd:C01313	(S)-N-[3-(3
KEGG	235.14	88.8	cpd:C05204	(S)-Tetrahydroprotoberberine
KEGG	308.1	106.84	cpd:C20129	(S)-Warfarin
KEGG	207.03	106.24	cpd:C21068	[5-(Aminomethyl)furan-3-yl]methyl phosphate
KEGG	294.18	1136.59	cpd:C10462	[6]-Gingerol 4 5 6-tetramethoxyphenyl)-2-propen-1-one
KEGG	276.2	1155.79	cpd:C10494	[6]-Shogaol
KEGG	146.05	1788.41	cpd:C14473	1(2H)-Phthalazinone

KEGG	464	74.15	cpd:C14895	1-(2-Hydroxy-3		
KEGG	338.07	91.52	cpd:C04905	1-(4-Amino-2-methylpyrimid-5-ylmethyl)-2-methylpyridinium bromide		
KEGG	454.1	85.81	cpd:C04823	1-(5'-Phosphoribosyl)-5-amino-4-(N-succinocarboxamide)-imidazole		
KEGG	366.1	88.87	cpd:C04734	1-(5'-Phosphor	7-dihydroxy-be	4-dioxocyclopenta[c][1]benzopyran-6-propanal
KEGG	338.05	625.45	cpd:C04437	1-(5-Phosphori	2	3-Trihydroxybenzene
KEGG	255.1	106.5	cpd:C04229	1-(Indol-3-yl)pr	2-Bis(4-nitrophenyl)ethane	
KEGG	348.08	117.28	cpd:C19591	4-Tetrahydro-a	2-Bis(chloromethoxy)ethane	
KEGG	126.03	4.58	cpd:C01108	1	2-Di-(9Z	15Z-octadecatrienoyl)-3-O-beta-D-galactosyl-sn-glycerol
KEGG	272.08	85.67	cpd:C14589	1	6-Dimethoxypyrene	
KEGG	157.99	78.76	cpd:C19348	1	8-Diazacyclotet	9-dione
KEGG	774.53	1480.32	cpd:C13871	1	D-galactosyl-sn-glycerol	
KEGG	262	131.12	cpd:C18260	1	11-Epoxy-3-geranylgeranylindole	
KEGG	226.17	9.07	cpd:C04277	1	16-Dihydroxyhexadecanoic acid	
KEGG	268.07	96.06	cpd:C15110	1-[6-Hydroxy-2-(4-hydroxyphenyl)-1-benzofuran-3-yl]ethanone		
KEGG	405.3	978.14	cpd:C20526	10	Cytidine diphosphate	
KEGG	288.23	937.8	cpd:C08285	10	D-Mannitol	
KEGG	566.22	656.05	cpd:C12405	100-1	polyketide products	
KEGG	468.1	670.76	cpd:C12403	104-1		
KEGG	294.17	968.99	cpd:C11635	10-Deoxysarpagine		
KEGG	392.1	112.64	cpd:C09447	10-epi-Eupator	4-dien-3-one acetate	
KEGG	473	1732.62	cpd:C00234	10-Formyltetrahydrofolate		
KEGG	364.1	84.5	cpd:C17939	10-Hydroxycan	17beta-Dihydr	4-dien-3-one
KEGG	314.19	1179.33	cpd:C14999	10-Hydroxyest	20-dione	
KEGG	422.14	1007.04	cpd:C11660	10-Hydroxymorroniside		
KEGG	316.2	1066.51	cpd:C14887	11alpha		
KEGG	344.24	992.98	cpd:C14964	11alpha-Hydroxy-12alpha-methyl-pregn-4-ene-3		
KEGG	338.23	1383.29	cpd:C15338	17beta-Dihydroxy-9alpha-fluoro-17alpha-methyl-5alpha-androstan-3-one		
KEGG	368.22	1014.55	cpd:C05964	11-Dehydro-thromboxane B2		
KEGG	322.08	99.18	cpd:C18682	11-Deoxylandomycinone		
KEGG	556.22	769.08	cpd:C11666	11-Hydroxyiridodial glucoside pentaacetate		
KEGG	480.09	618.1	cpd:C17520	11-o-Galloylbe	20-dione	
KEGG	440.37	1585.68	cpd:C19943	11-Oxo-beta-ar	6-dienoate	
KEGG	410.14	133.85	cpd:C10464	12a-Hydroxyrotenone		
KEGG	408.13	160.55	cpd:C14669	12alpha-Bromo-11beta-hydroxypregn-4-ene-3		
KEGG	386.2	369.86	cpd:C15569	12alpha-Hydroxy-3-oxochola-4		
KEGG	532.2	778.46	cpd:C08767	12alpha-Hydroxyamoorstatin		
KEGG	242.2	1176.7	cpd:C16665	12-Methyltetradecanoic acid		
KEGG	660.42	1297.07	cpd:C09153	12-O-Palmitoyl-16-hydroxyphorbol 13-acetate		
KEGG	212.14	1129.6	cpd:C16311	12-Oxo-9(Z)-dodecenoic acid		
KEGG	614.24	677.84	cpd:C11829	13(1)-Hydroxy-magnesium-protoporphyrin IX 13-monomethyl ester		
KEGG	346.23	1155.06	cpd:C04170	13-(2-Methylcrotonoyl)oxylupanine		
KEGG	258.2	1397.19	cpd:C20696	13-Apo-beta-ca	20-dione 7-acetate	
KEGG	518.38	1116.25	cpd:C04103	13-beta-D-Glucosyloxydocosanoate		
KEGG	412.37	1503.32	cpd:C05108	14-Demethyllanosterol		
KEGG	404	1744.34	cpd:C14922	15beta-Hydrox	17beta-Dihydroxyandrost-4-en-3-one dipropionate	
KEGG	555	1733	cpd:C20689	15-Demethylaclacinomycin T		
KEGG	598.24	1493.71	cpd:C20667	15-Oxo-beta-bilirubin		
KEGG	416.3	1457.82	cpd:C15289	16alpha		
KEGG	306.2	1041.64	cpd:C15074	16alpha-Fluoro-17alpha-hydroxyandrost-4-en-3-one		
KEGG	322.17	980.97	cpd:C15004	16beta-Chloro-17beta-hydroxyandrost-4-en-3-one		

KEGG	366.19	234.52	cpd:C11675	16-Methoxytabersonine
KEGG	340.25	1442.74	cpd:C14998	17beta-(1H-Imidazol-4-yl)androst-5-en-3beta-ol
KEGG	270.16	1115.57	cpd:C14485	17beta-Dihydroequilin
KEGG	448.21	990.69	cpd:C11237	17beta-Estradiol 17-(beta-D-glucuronide)
KEGG	528.17	770.89	cpd:C11289	17beta-Estradi 17-dimethyl-5alpha-androstan-3-one
KEGG	334.25	1177.07	cpd:C15137	17beta-Hydroxy-2alpha-(hydroxymethyl)-17-methyl-5alpha-androstan-3-one
KEGG	348.27	1440.49	cpd:C14919	17beta-Hydroxy-2alpha-(methoxymethyl)-17-methyl-5alpha-androstan-3-one
KEGG	318.3	1103.93	cpd:C15099	17beta-Hydrox 4-dieno[2 3-d]isoxazol-17beta-ol
KEGG	376.2	802.87	cpd:C14973	17beta-Hydroxy-7alpha-mercaptoandrost-4-en-3-one 7-propionate
KEGG	274.19	1058.04	cpd:C15257	17beta-Hydroxyestr-5(10)-en-3-one
KEGG	327.2	1115.8	cpd:C15177	17-Methylandrosta-2
KEGG	368.21	994.02	cpd:C15985	17-O-Acetylajmaline
KEGG	316.28	1066.54	cpd:C14933	17-Propyl-5alpha-androst-2-en-17beta-ol
KEGG	334.1	750.88	cpd:C12400	19-Hydroxy-8-O-methyltetrangulol
KEGG	272.18	1164.63	cpd:C14500	19-Norandrostenedione
KEGG	259.05	89.97	cpd:C01283	1-Amino-1-deoxy-scyllo-inositol 4-phosphate
KEGG	101.05	109.32	cpd:C01234	1-Aminocyclopropane-1-carboxylate
KEGG	111.01	1786.62	cpd:C11033	1-Aminomethylphosphonic acid
KEGG	342.1	93.95	cpd:C10433	1-Caffeoyl-beta-D-glucose
KEGG	332.1	112.22	cpd:C15387	1-Dehydro-9-fluoro-11-oxotestololactone
KEGG	162.08	143.58	cpd:C13840	1-Ethyl-2-benzimidazolinone
KEGG	836.54	1476.8	cpd:C13888	1-Hexadecanoyl-2-(9Z-octadecenoyl)-sn-glycero-3-phospho-1'-myo-inositol
KEGG	744.59	1719.31	cpd:C13876	1-Hexadecano 2-dideoxy-sn-glycero-3-phosphocholine
KEGG	828.5	1740.03	cpd:C13885	1-Hexadecanoyl-2-(9Z-octadecenoyl)-sn-glycero-3-phospho-sn-glycerol 3'-phosphate
KEGG	754.5	1515.59	cpd:C13890	1-Hexadecanoyl-2-(9Z-octadecenoyl)-sn-glycero-3-pyrophosphate
KEGG	735.58	1387.15	cpd:C04873	1-Hexadecylthio-2-hexadecanoylamino-1
KEGG	552.43	1590.17	cpd:C15860	1'-Hydroxytorulene
KEGG	384.1	92.84	cpd:C20503	1'-Hydroxyversicolorone
KEGG	139	103.96	cpd:C16697	1-Indolizidinone
KEGG	410.2	201.01	cpd:C10071	1-Isomangostir 4-tetrahydro-6-methoxy-2-naphthyl)-2(1H)-pyridone
KEGG	520.34	1205.06	cpd:C04100	1-Linoleoylglycerophosphocholine
KEGG	232.1	952.74	cpd:C18259	1-Methoxypyrene
KEGG	269	1723.94	cpd:C14896	1-Methyl-6-(1
KEGG	137.07	106.58	cpd:C02918	1-Methylnicotinamide
KEGG	84.1	1669.42	cpd:C06178	1-Methylpyrrolinium
KEGG	182.04	84.15	cpd:C16359	1-Methyluric acid
KEGG	463	72.81	cpd:C19761	1-O-(2-Acetamido-2-deoxy-alpha-D-glucopyranosyl)-1D-myo-inositol 3-phosphate
KEGG	356.1	265.02	cpd:C17759	1-O-Feruloyl-beta-D-glucose
KEGG	496.3	1176.91	cpd:C04102	1-Palmitoylglycerophosphocholine
KEGG	274.01	88.29	cpd:C04037	1-Phospho-alpha-D-galacturonate
KEGG	145.05	160.68	cpd:C06415	2(1H)-Quinolinone
KEGG	316.1	126.23	cpd:C14893	2-(4-Chlorophenyl)-3-phenyl-3-(2-pyridinyl)acrylonitrile
KEGG	334.07	1092.8	cpd:C04641	2-(alpha-D-Galactosyl)-sn-glycerol 3-phosphate
KEGG	469.1	665.32	cpd:C05125	2-(alpha-Hydroxyethyl)thiamine diphosphate2,36'-Heptabromodiphenyl ether
KEGG	391.1	112.51	cpd:C21374	2-(Glutathion-S 2-Bis(4-hydroxyphenyl)hexafluoropropane
KEGG	155.99	90.72	cpd:C03920	2-(Methylthio)ethanesulfonate
KEGG	715.4	1196.12	cpd:C18139	2 3 4 6'-Peptahydroxychalcone 4'-O-glucoside
KEGG	336.1	1130.82	cpd:C14350	2 3 4-Trichlorobiphenyl
KEGG	380	72.49	cpd:C15050	2-Dimethyl-3-[4-(acetyloxy)phenyl]-4-ethyl-2I 10-Tetrahydroxyberberine
KEGG	450.1	366.38	cpd:C16408	2' 3'-Cyclic UMP

KEGG	255.96	80.05	cpd:C14357	2 3-Dimethylmaleate
KEGG	296.1	105.61	cpd:C12331	2 4-Hexadienal
KEGG	306.03	1738.5	cpd:C02355	2' 5-Diamino-6-(5-phospho-D-ribosylamino)pyrimidin-4(3H)-one
KEGG	144.04	3.76	cpd:C00922	2 7 4'-Trihydroxyisoflavanone
KEGG	96.1	1667.09	cpd:C19249	2 21-Diprenylterpendole J
KEGG	353.1	84.75	cpd:C01304	2,5-Diamino-6-(5-phospho-D-ribosylamino)pyrimidin-4(3H)-one
KEGG	272.07	679.55	cpd:C15567	2,7,4'-Trihydroxyisoflavanone; 2,4',7-Trihydroxyisoflavanone
KEGG	657.4	1353.99	cpd:C20594	20,21-Diprenylterpendole
KEGG	480.3	1239.87	cpd:C02633	20-Hydroxyecdysone
KEGG	455.2	603.04	cpd:C03577	20-Hydroxyleul 3alpha-(Difluoromethylene)-5alpha-androstan-17beta-ol acetate
KEGG	491.2	627.47	cpd:C20135	20-O-Methyl-1 11 20-trione
KEGG	581.36	1267.61	cpd:C20760	20-Oxo-5-O-be 5-dihydroxy-6-oxo-7-(phosphooxy)heptanoate
KEGG	366.24	1440.76	cpd:C15322	2alpha
KEGG	342.22	1146.25	cpd:C15301	2alpha-Methylpregn-4-ene-3
KEGG	287.04	810.29	cpd:C20644	2-Amino-4
KEGG	225.13	990.87	cpd:C20698	2-Aminoethyl diphenylborinate
KEGG	125.02	76.82	cpd:C03557	2-Aminoethylphosphonate
KEGG	173.07	95.57	cpd:C21248	2-Aminovalienone
KEGG	175.09	91.51	cpd:C10898	2-Benzimidazolylguanidine
KEGG	171.95	1787.62	cpd:C14841	2-Bromophenc 6-dideoxy-6-sulfo-D-gluconate
KEGG	276.02	91.03	cpd:C04234	2-Carboxy-D-arabinitol 1-phosphate
KEGG	187.99	1720.47	cpd:C18304	2-Chloro-5-methyl-cis-dienelactone
KEGG	242.01	108.79	cpd:C21180	2-Dehydro-3
KEGG	482.22	776.55	cpd:C20509	2'-Dehydrokanamycin A
KEGG	146.06	84.9	cpd:C00966	2-Dehydropantoate
KEGG	272.03	103.61	cpd:C17692	2-epi-5-epi-Valiolone 7-phosphate
KEGG	278.15	1270.57	cpd:C03343	2-Ethylhexyl pł 4-benzoquinone
KEGG	111.05	87.12	cpd:C11010	2-Fluoroaniline 4-pentadienoate
KEGG	259.2	1396.96	cpd:C11848	2-Heptyl-3-hydroxy-quinolone
KEGG	124.02	77.37	cpd:C07103	2-Hydroxy-1
KEGG	114.03	56.28	cpd:C00596	2-Hydroxy-2,4-pentadienoate
KEGG	157.07	104.42	cpd:C17361	2-Hydroxyethylclavam
KEGG	148.04	89.33	cpd:C02630	2-Hydroxyglutarate
KEGG	468.4	1510.39	cpd:C02793	2-Hydroxy-vita 6-N-Bis(2 3-dihydroxybenzoyl)-L-lysine
KEGG	236.04	105.06	cpd:C19688	2-Imino-3-(7-chloroindol-3-yl)propanoate
KEGG	286.06	134.17	cpd:C15439	2-Methacryloyloxyethyl phenyl phosphate
KEGG	418.1	138.27	cpd:C06446	2-N,6-N-Bis(2,3-dihydroxybenzoyl)-L-lysine
KEGG	582.3	1377.82	cpd:C06512	2'-Norberbamunine
KEGG	372.07	1002.59	cpd:C03062	2-O-Caffeoylglucarate
KEGG	162.04	1780.34	cpd:C17211	2-Oxo-5-methylthiopentanoic acid
KEGG	190.07	109.39	cpd:C17220	2-Oxo-7-methylthioheptanoic acid
KEGG	204.08	160.41	cpd:C17224	2-Oxo-8-methylthiooctanoic acid
KEGG	188.07	161.29	cpd:C16589	2-Oxosuberate
KEGG	212.01	110.32	cpd:C17947	2-Phosphinomethylmalate
KEGG	229.2	1404.72	cpd:C13795	2S-Amino-tridecanoic acid
KEGG	365.1	84.41	cpd:C14862	2-S-Glutathion 4-Cyclopentadien-1-ylidene)pregn-4-en-20-one
KEGG	422.07	95.1	cpd:C20985	2-Sulfotrehalos 4-Dihydroxypyridin-1-yl)-L-alanine
KEGG	614.4	1343.88	cpd:C11321	3-((3-Cholamidopropyl)dimethylammonium)-1-propanesulfonate
KEGG	362.3	1349.23	cpd:C14968	3-(2 5-diene-11 17-dione
KEGG	199.07	113.88	cpd:C04446	3-(3,4-Dihydroxypyridin-1-yl)-L-alanine

KEGG	180.04	113.97	cpd:C01179	3-(4-Hydroxypl	17beta-Diaceto	3	5(10)-trien-6-one	
KEGG	374.2	1383.31	cpd:C14992	3-(Acetyloxy)-9	3'-Dimethylbenzidine			
KEGG	444.3	1322.79	cpd:C04590	3-(O-Geranylge	4	6-Trichlorocatechol		
KEGG	370.2	724.25	cpd:C15313	3	4-Dihydroanhydrorhodovibrin			
KEGG	212.13	967.99	cpd:C14443	3	4-Dihydroxy-2-	6E)-farnesyl]-3	4-dihydroquinoline 1-oxide	
KEGG	211.92	1790.49	cpd:C12831	3	4-Dihydroxy-L-phenylalanine			
KEGG	568	1743.76	cpd:C15886	3	5	3'-Triiodothyropyruvate		
KEGG	397	976.02	cpd:C21142	3	5	7	9	13-Pentadecen-2-one
KEGG	197	1794.91	cpd:C00355	3	5-Dihydroxy-1	4-naphthoquinone		
KEGG	650	1743.87	cpd:C03832	3	5-Diiodo-L-tyrosine			
KEGG	214.14	924.75	cpd:C21360	3	5-Dinitro-L-tyrosine			
KEGG	190.03	93.61	cpd:C04110	3	d]pyran-6-one			
KEGG	433	1720.3	cpd:C01060	4	d]pyran-6-one-tyrosine			
KEGG	271.04	152.61	cpd:C03225	3	12alpha-Dihydroxy-5beta-chol-6-enoate			
KEGG	333.1	116.34	cpd:C14951	3-[(2-Chlorobe	7alpha	12alpha-Trihydroxy-5beta-cholestane		
KEGG	456.36	1391.51	cpd:C20510	30-Hydroxy-11-oxo-beta-amyrin				
KEGG	390.28	1606.38	cpd:C11637	3alpha				
KEGG	420.36	1515.76	cpd:C05454	3alpha,7alpha,12alpha-Trihydroxy-5beta-cholestane				
KEGG	470.34	1060.92	cpd:C03930	3alpha-Hydroxyglycyrrhetinate				
KEGG	308.19	1013.07	cpd:C15396	3beta-Chloro-5	2-dihydroxycyc	5-diene		
KEGG	444.36	1369.69	cpd:C04840	3beta-Hydroxy-4beta-methyl-5alpha-cholest-7-ene-4alpha-carboxylate				
KEGG	258.05	118.46	cpd:C19357	3-Carbethoxypsoralen				
KEGG	146.01	94	cpd:C12837	3-Chloro-cis-1				
KEGG	247.04	86.4	cpd:C11290	3-Chlorosalicylanilide				
KEGG	484.2	844.05	cpd:C21254	3''-Deamino-3''-hydroxykanamycin B				
KEGG	446.34	1506.64	cpd:C15792	3-Dehydroteasterone				
KEGG	256.18	1055.44	cpd:C14239	3-Deoxyestradi	7	8-trimethoxy-2-naphthoate		
KEGG	186	89.52	cpd:C02514	3-Fumarylpyruvate				
KEGG	100	94.9	cpd:C08492	3-Hexenol				
KEGG	278.08	87.9	cpd:C21322	3-Hydroxy-6	N6	N6-trimethyl-L-lysine		
KEGG	566.41	1594.3	cpd:C15965	3'-Hydroxyechinenone				
KEGG	526.12	118.66	cpd:C11462	3-Hydroxy-L-ty	3	5(10)	16-tetraene	
KEGG	205.16	1287.18	cpd:C01259	3-Hydroxy-N6	3	5(10)-triene-16	17-dione 16-oxime	
KEGG	297.27	1082.05	cpd:C06121	3-Ketosphingosine				
KEGG	268.2	1242.63	cpd:C15107	3-Methoxyestra-1				
KEGG	313.17	1278.18	cpd:C15078	3-Methoxyestra-1				
KEGG	360.13	152.86	cpd:C17754	3-Methoxytyramine-betaxanthin				
KEGG	84.06	110.49	cpd:C07330	3-Methyl-2-butenal				
KEGG	118.02	108.27	cpd:C11119	3-Nitrosothiazolidine				
KEGG	546.34	1018.33	cpd:C06630	3-O-alpha-Mycarosylerythronolide B				
KEGG	214.16	933.77	cpd:C02367	3-Oxododecanoic acid				
KEGG	153.01	108.79	cpd:C00606	3-Sulfino-L-alar	21-Dehydrocorynantheine aldehyde			
KEGG	136.05	100.96	cpd:C07085	3-Vinylcatecho	4'-Diapolycopenedial			
KEGG	372.03	73.54	cpd:C13677	4-(N-Maleimido	4'-Diapophytoene			
KEGG	351	1720.77	cpd:C11679	4	4'-Diapophytofluene			
KEGG	428.27	1094.42	cpd:C19798	4	4'-Dihydroxy-3	5-dimethoxydihydrostilbene		
KEGG	408.38	1337	cpd:C16144	4	4'-Methylene bis(2-methylaniline)			
KEGG	406.36	1643.89	cpd:C19840	4	4'-Stilbenedicarboxamidine dihydrochloride			
KEGG	274.12	822.49	cpd:C10256	4	5-Chrysenedicarboxylate			
KEGG	226.15	350.17	cpd:C19260	4,4'-Methylene bis(2-methylaniline)				

KEGG	336.09	84.79	cpd:C15389	4,4'-Stilbenedicarboxamidine dihydrochloride
KEGG	316.07	178.49	cpd:C18277	4,5-Chrysenedicarboxylate
KEGG	219.04	87.34	cpd:C04556	4-Amino-2-methyl-5-(phosphooxymethyl)pyrimidine
KEGG	138.09	108.05	cpd:C20267	4-Amino-5-ami 4' 5' 6'-tetramethoxychalcone
KEGG	103.1	86.99	cpd:C00334	4-Aminobutanoate
KEGG	87.07	62.32	cpd:C00555	4-Aminobutyra 5-cyclohexadie 2-dione
KEGG	450.17	682.06	cpd:C15080	4-Benzyloxy-2'-hydroxy-3'
KEGG	430.38	1668.38	cpd:C04814	4beta-Hydroxymethyl-4alpha-methyl-5alpha-cholest-7-en-3beta-ol
KEGG	185.93	1720.4	cpd:C14846	4-Bromo-3
KEGG	188.04	1783.28	cpd:C06584	4-Chlorobiphenyl
KEGG	126.02	109.13	cpd:C14451	4-Chlorotoluene
KEGG	108.1	1663.58	cpd:C01468	4-Cresol
KEGG	208.15	845.31	cpd:C14236	4-Heptyloxyphenol
KEGG	381.08	84.67	cpd:C06628	4-Hydroxy-6-methylpretetramide
KEGG	120.06	108.09	cpd:C05627	4-Hydroxystyrene
KEGG	598.4	1123.32	cpd:C15928	4-Ketomyxol
KEGG	478.1	593.16	cpd:C08423	4-Methoxyglucobrassicin
KEGG	314.08	223.06	cpd:C17784	4'-Methylcapillarisin
KEGG	148.02	1274.21	cpd:C01180	4-Methylthio-2 2'-bithiophene
KEGG	352.08	88.14	cpd:C11584	4-Methylumbe 10-Methenyltetrahydrofolate
KEGG	183.05	77.62	cpd:C00847	4-Pyridoxate 11a-Dehydrotetracycline
KEGG	216.01	91.52	cpd:C08397	5-(3-Buten-1-ynyl)-2
KEGG	456.2	701.59	cpd:C00445	5,10-Methenyltetrahydrofolate
KEGG	442.1	654.98	cpd:C03206	5a,11a-Dehydrotetracycline
KEGG	285.02	78.93	cpd:C05155	5-Amino-4-chloro-2-(5-hydroxymuconoyl)-3(2H)-pyridazinone
KEGG	127.04	90.4	cpd:C05516	5-Amino-4-imidazole carboxylate
KEGG	83.05	97.49	cpd:C05239	5-Aminoimidazole
KEGG	418.09	746.57	cpd:C06435	5'-Butyrylphosphoinosine
KEGG	394.08	93.31	cpd:C06436	5'-Butyrylphosphouridine
KEGG	216.03	85.79	cpd:C04052	5-Carboxy-2-oxohept-3-enedioate
KEGG	245.08	109.23	cpd:C16635	5'-Deoxy-5-fluorocytidine
KEGG	138.02	112.94	cpd:C14480	5-Diazouracil
KEGG	472.18	616.16	cpd:C00664	5-Formiminotetrahydrofolate
KEGG	219.1	120.92	cpd:C05646	5-Hydroxyindolepyruvate
KEGG	220.1	118.34	cpd:C00643	5-Hydroxy-L-tryptophan
KEGG	252.07	664.1	cpd:C05648	5-Hydroxy-N-formylkynurenine
KEGG	232.04	86.98	cpd:C02951	5-Hydroxyxanthotoxin
KEGG	205.1	92.2	cpd:C05660	5-Methoxyindoleacetate
KEGG	142.04	85.53	cpd:C05281	5-Methylbarbiturate
KEGG	297.09	200.33	cpd:C00170	5'-Methylthioadenosine
KEGG	180.05	123.38	cpd:C03089	5-Methylthio-D-ribose
KEGG	331.13	106.6	cpd:C03996	5'-O-beta-D-Gl 8a-Seco-6 8a-deoxy-5-oxoavermectin ''2b'' aglycone
KEGG	308.2	1023.42	cpd:C10373	5-O-Methylem 8-Di-DMA-chrysin
KEGG	314.05	101.42	cpd:C04376	5'-Phosphoribosyl-N-formylglycinamide
KEGG	572.3	918.76	cpd:C11953	6 5-diaminopyrazine-3-carboxamide
KEGG	390.18	787.75	cpd:C11573	6,8-Di-DMA-chrysin
KEGG	466	78.67	cpd:C15803	6alpha-Hydrox 3-dimethylben 4-diol
KEGG	257.08	115.57	cpd:C13753	6-Chloro-3
KEGG	260.02	94.12	cpd:C21182	6-Deoxy-6-sulfo-D-gluconate
KEGG	410.32	1515.53	cpd:C20738	6-Geranylgeranyl-2

KEGG	303.05	731.75	cpd:C08646	6-Hydroxycyan 12-Dimethylbenz[a]anthracene
KEGG	393.1	104.8	cpd:C16620	6-Methylthiogı 8-Dihydroxykynurenate
KEGG	494.26	627.3	cpd:C21268	6'-Oxo-G418
KEGG	256.13	264.24	cpd:C19488	7,12-Dimethylbenz[a]anthracene
KEGG	221.03	88.53	cpd:C01111	7,8-Dihydroxykynurenate
KEGG	335.11	106.27	cpd:C21163	7-Demethylmitomycin A
KEGG	360.14	153.59	cpd:C11636	7-Deoxyloganate
KEGG	165.07	116.63	cpd:C02242	7-Methylguani 8a-Deoxyoleandolide
KEGG	378.08	87.35	cpd:C03998	7-Methylguanosine 5'-phosphate
KEGG	371.2	723.17	cpd:C05322	7-O-Acetylsalutaridinol
KEGG	372.25	1189.61	cpd:C11989	8,8a-Deoxyoleandolide
KEGG	299.06	109.93	cpd:C11326	8-Anilino-1-naphthalene sulfonic acid
KEGG	418.32	1024.55	cpd:C06733	8'-apo-beta-Ca 11-dione
KEGG	498.2	668.7	cpd:C11658	8-Epiiridodial glucoside tetraacetate
KEGG	318.1	651.96	cpd:C12398	8-O-Methyltetrangulol
KEGG	394.11	923.15	cpd:C15371	9-Bromo-17beta-hydroxy-17-methylandrost-4-ene-3
KEGG	376.28	1638.61	cpd:C20692	9-cis-10'-Apo-beta-carotenal
KEGG	203.13	1205.2	cpd:C17253	9-Methylthiononanaldoxime
KEGG	426.19	695.59	cpd:C15970	Abscisic acid glucose ester
KEGG	151.06	109.55	cpd:C06804	Acetaminophen
KEGG	60.02	112.57	cpd:C00033	Acetate
KEGG	324.11	91.84	cpd:C06806	Acetohexamide
KEGG	313.13	94.22	cpd:C08513	Acetylcaranine
KEGG	811.3	852.97	cpd:C18638	Aclacinomycin A
KEGG	311.12	106.17	cpd:C10629	Acronidine
KEGG	443.01	77.62	cpd:C13743	Adenosine 5-O-(3-thiodiphophate)
KEGG	550.28	1156.08	cpd:C08843	Adonitoxin
KEGG	582.41	1382.23	cpd:C15968	Adonixanthin
KEGG	500.34	1237.99	cpd:C09993	Adouetine X
KEGG	298.08	93.44	cpd:C10199	Afrormosin
KEGG	546.2	643.19	cpd:C10209	Afzelechin-(4alpha->8)-afzelechin
KEGG	474.2	633.13	cpd:C10671	Agrimophol
KEGG	399.1	105.69	cpd:C18414	Alanycarb
KEGG	480.16	642.62	cpd:C17457	Albiflorin
KEGG	256.12	910.21	cpd:C20520	Albonoursin
KEGG	388.15	816.55	cpd:C09059	Alectrol
KEGG	472.36	1197.59	cpd:C17460	Alisol B
KEGG/MASSBANK	173.06	93.66	cpd:C19043	Allidochlor
KEGG/MASSBANK	622.27	1428.75	cpd:C05346	Allosamidine 5-L-Arabinotriose
KEGG/MASSBANK	654.38	1193.46	cpd:C04216	all-trans-Heptaprenyl diphosphate
KEGG/MASSBANK	564.18	89.43	cpd:C17779	Aloinoside A
KEGG/MASSBANK	414.14	609.89	cpd:C20571	alpha-1
KEGG/MASSBANK	566.27	981.29	cpd:C08847	alpha-Antiarin
KEGG/MASSBANK	110.01	1789.11	cpd:C18676	alpha-Chlorohydrin
KEGG/MASSBANK	387.2	370.39	cpd:C16544	alpha-Hydroxytamoxifen
KEGG/MASSBANK	118.08	90.03	cpd:C14395	alpha-Methylstyrene
KEGG/MASSBANK	308.08	722.76	cpd:C06817	Alprazolam
KEGG/MASSBANK	716.4	1204.6	cpd:C08433	Amataine
KEGG/MASSBANK	536.3	1177.64	cpd:C07773	Ambenonium
KEGG/MASSBANK	220.05	100.75	cpd:C11490	AMCC

KEGG/MASSBANK	59.04	1757.37	cpd:C06735	Aminoacetaldehyde
KEGG/MASSBANK	73.05	1761.96	cpd:C01888	Aminoacetone
KEGG/MASSBANK	293.19	1133.54	cpd:C10995	Amitraz
KEGG/MASSBANK	435.24	682.04	cpd:C12342	Ancistrobrevine A
KEGG/MASSBANK	426.1	1032.91	cpd:C02811	Anhydrotetracycline
KEGG/MASSBANK	275.2	1057.04	cpd:C09852	Annotine
KEGG/MASSBANK	584.42	1383.5	cpd:C08579	Antheraxanthin
KEGG/MASSBANK	207.08	428.3	cpd:C15549	Anthocyanin
KEGG/MASSBANK	208.05	1779.7	cpd:C16207	Anthraquinone
KEGG/MASSBANK	270.05	886.83	cpd:C01477	Apigenin
KEGG/MASSBANK	564.15	744.58	cpd:C04858	Apigenin 7-O-[beta-D-apiosyl-(1->2)-beta-D-glucoside]
KEGG/MASSBANK	578.16	687.69	cpd:C12627	Apigenin 7-O-neohesperidoside
KEGG/MASSBANK	294.06	718.22	cpd:C17132	Aplysin
KEGG/MASSBANK	582.28	633.23	cpd:C09999	Aralionine A
KEGG/MASSBANK	426.2	189.46	cpd:C09116	Archangelicin
KEGG/MASSBANK	744.48	1737.04	cpd:C20094	Argiopinin II
KEGG/MASSBANK	659.42	1297.05	cpd:C20095	Argiopinin III
KEGG/MASSBANK	306.15	148.36	cpd:C09299	Arnicolide A
KEGG/MASSBANK	488.35	1053.73	cpd:C08617	Asiatic acid
KEGG/MASSBANK	340.07	95.05	cpd:C17400	Asparenomycin A
KEGG/MASSBANK	294.12	200.39	cpd:C11045	Aspartame
KEGG/MASSBANK	582.2	850.16	cpd:C11655	Asperuloside tetraacetate
KEGG/MASSBANK	296.07	1120.92	cpd:C02006	Aspulvinone E
KEGG/MASSBANK	406.13	107.53	cpd:C10245	Astringin
KEGG/MASSBANK	216.15	915.09	cpd:C16919	Atractylone
KEGG/MASSBANK	576.3	951.6	cpd:C17866	Atractyloside I
KEGG/MASSBANK	287.06	133.18	cpd:C08575	Aurantinidin
KEGG/MASSBANK	492.13	768.2	cpd:C10303	Aurantio-obtusin beta-D-glucoside
KEGG/MASSBANK	556.14	772.8	cpd:C08995	Aurasperone D
KEGG/MASSBANK	448.1	615.65	cpd:C16409	Aureusidin 6-O-glucoside
KEGG/MASSBANK	450.15	115.45	cpd:C09478	Auriculoside
KEGG/MASSBANK	685.4	1272.82	cpd:C08661	Avadharidine
KEGG/MASSBANK	584.33	1024.29	cpd:C11964	Avermectin A1b aglycone
KEGG/MASSBANK	732.41	1347.61	cpd:C11957	Avermectin B2b monosaccharide
KEGG/MASSBANK	381.16	115	cpd:C07768	Azelastine
KEGG/MASSBANK	748.51	1736.46	cpd:C06838	Azithromycin
KEGG/MASSBANK	270.12	953.39	cpd:C20942	Bacilysin
KEGG/MASSBANK	446.1	661.54	cpd:C10025	Baicalin
KEGG/MASSBANK	520.22	819.09	cpd:C07813	Beclomethasone dipropionate
KEGG/MASSBANK	322.98	77.29	cpd:C18907	Benodanil
KEGG/MASSBANK	410.09	638.69	cpd:C10937	Bensulfuron-methyl
KEGG/MASSBANK	381.15	113.87	cpd:C18415	Benthiavalicarb isopropyl
KEGG/MASSBANK	118.05	125.88	cpd:C02009	Benzimidazole
KEGG/MASSBANK	226.08	101.4	cpd:C19342	Benzo[ghi]fluoranthene
KEGG/MASSBANK	430.1	209.48	cpd:C18555	Benzofenap
KEGG/MASSBANK	118.04	949.14	cpd:C14512	Benzofuran
KEGG/MASSBANK	103.04	87	cpd:C09814	Benzonitrile
KEGG/MASSBANK	363.09	251.75	cpd:C19021	Benzoximate
KEGG/MASSBANK	234.15	1781.63	cpd:C02253	Benzoylagmatine
KEGG/MASSBANK	366.27	1298.96	cpd:C06847	Bepridil

KEGG/MASSBANK	608.29	1556.32	cpd:C09357	Berbamine
KEGG/MASSBANK	352.12	125.85	cpd:C09360	Berberastine
KEGG/MASSBANK	162.09	111.6	cpd:C08234	beta-Cymaropyranose
KEGG/MASSBANK	532.41	1591.45	cpd:C16340	beta-Isorenieratene
KEGG/MASSBANK	388.09	109.48	cpd:C08539	Betanidin
KEGG/MASSBANK	550.14	89.84	cpd:C08540	Betanin
KEGG/MASSBANK	119.02	90.89	cpd:C05669	beta-Nitropropanoate
KEGG/MASSBANK	428.1	636.69	cpd:C10730	beta-Peltatin A methyl ether
KEGG/MASSBANK	233.08	105.49	cpd:C19347	Bis(1-aziridinyl)morpholinophosphine sulfide
KEGG/MASSBANK	352.03	109	cpd:C14346	Bisphenol A bis(chloroformate)
KEGG/MASSBANK	264.11	113.51	cpd:C17967	Bluensidine
KEGG/MASSBANK	370.32	1134.64	cpd:C15119	B-Norcholest-4-en-3-one
KEGG/MASSBANK	266.14	1357.87	cpd:C09052	Borreline
KEGG/MASSBANK	406.11	674.8	cpd:C14647	Braxoron
KEGG/MASSBANK	283.13	1775.21	cpd:C20563	Brevianamide F
KEGG/MASSBANK	573.2	684.13	cpd:C13851	BRL 32872
KEGG/MASSBANK	522.08	83.93	cpd:C18694	Brodifacoum
KEGG/MASSBANK	548.2	760.54	cpd:C08749	Bruceantin
KEGG/MASSBANK	682	77.44	cpd:C08753	Bruceoside A
KEGG/MASSBANK	273.09	85.84	cpd:C10849	Brugine
KEGG/MASSBANK	374.14	284.87	cpd:C09351	Budlein A
KEGG/MASSBANK	288.22	922.81	cpd:C07529	Bupivacaine
KEGG/MASSBANK	385.25	1014.59	cpd:C06861	Buspirone
KEGG/MASSBANK	88.05	694.44	cpd:C00246	Butanoic acid
KEGG/MASSBANK	226.12	913.94	cpd:C19142	Butopyronoxyl
KEGG/MASSBANK	596.17	701.6	cpd:C09616	Butrin
KEGG/MASSBANK	470.19	118.48	cpd:C08997	Butyrylmallotochromene
KEGG/MASSBANK	306.17	1005.52	cpd:C01670	Bz-Arg-OEt
KEGG/MASSBANK	194	1794.01	cpd:C07481	Caffeine
KEGG/MASSBANK	99.95	82.57	cpd:C08129	Calcium carbonate
KEGG/MASSBANK	109.9	85.43	cpd:C08130	Calcium chloride anhydrous
KEGG/MASSBANK	270.04	876.28	cpd:C18380	Calcium levulinate anhydrous
KEGG/MASSBANK	416.16	587.09	cpd:C09158	Calophyllolide
KEGG/MASSBANK	348.11	86.38	cpd:C01897	Camptothecin
KEGG/MASSBANK	606.24	1351.5	cpd:C09370	Cancentrine
KEGG/MASSBANK	440.16	188.96	cpd:C07468	Candesartan
KEGG/MASSBANK	596.2	655.07	cpd:C17906	Cannabisin B
KEGG/MASSBANK	624.2	91.5	cpd:C17908	Cannabisin D
KEGG/MASSBANK	359.1	96.94	cpd:C12650	Capecitabine
KEGG/MASSBANK	236.18	1086.44	cpd:C09627	Capsidiol
KEGG/MASSBANK	428.19	624.83	cpd:C09106	Carapanaubine
KEGG/MASSBANK	61.02	78.32	cpd:C01563	Carbamate
KEGG/MASSBANK	221.11	1716.12	cpd:C14291	Carbofuran
KEGG/MASSBANK	580.14	248.8	cpd:C10026	Carlinoside
KEGG/MASSBANK	213.01	111.3	cpd:C06873	Carmustine
KEGG/MASSBANK	330.18	1160.21	cpd:C09069	Carnosol
KEGG/MASSBANK	189.1	98.93	cpd:C02256	Castanospermine
KEGG/MASSBANK	482.1	682.99	cpd:C09775	Catalposide
KEGG/MASSBANK	398.13	692.72	cpd:C17097	Caulerpin
KEGG/MASSBANK	204.02	89.57	cpd:C11164	CCCP

KEGG/MASSBANK	488.1	119.2	cpd:C00307	CDP-choline
KEGG/MASSBANK	460.08	85	cpd:C03486	CDP-N-methylethanolamine
KEGG/MASSBANK	739.4	1154.97	cpd:C15822	CE-108
KEGG/MASSBANK	304.12	964.31	cpd:C09130	Ceceline
KEGG/MASSBANK	480.12	109.46	cpd:C08111	Cefepime
KEGG/MASSBANK	427.1	1073.1	cpd:C06887	Cefoxitin
KEGG/MASSBANK	514	1721.24	cpd:C11199	Cefpirome
KEGG/MASSBANK	410.04	114.57	cpd:C08117	Ceftibuten
KEGG/MASSBANK	379.23	1153.25	cpd:C10578	Celabenzine
KEGG/MASSBANK	606.27	1542.61	cpd:C09391	Cepharanthine
KEGG/MASSBANK	534.15	92.22	cpd:C10309	Cercosporin
KEGG/MASSBANK	223.12	1756.12	cpd:C12058	Cerulenin
KEGG/MASSBANK	349.21	719.76	cpd:C13786	Cetiedil
KEGG/MASSBANK	320.99	73.11	cpd:C14128	CGH 2466
KEGG/MASSBANK	184.13	916.87	cpd:C09633	Chamazulene
KEGG/MASSBANK	474.1	758	cpd:C10437	Chicoric acid
KEGG/MASSBANK	424.17	591.51	cpd:C01674	Chitobiose
KEGG/MASSBANK	414.04	110.84	cpd:C10943	Chlorimuron-ethyl
KEGG/MASSBANK	233.97	1789.64	cpd:C18686	Chlormephos
KEGG/MASSBANK	314.09	129.71	cpd:C09358	Chlorochrymorin
KEGG/MASSBANK	64.01	1724.82	cpd:C18248	Chloroethane
KEGG/MASSBANK	628.22	655.81	cpd:C16541	Chlorophyllide b
KEGG/MASSBANK	212.07	131.78	cpd:C18817	Chlorotoluron
KEGG/MASSBANK	390.1	108.48	cpd:C07780	Chlorpheniramine maleate
KEGG/MASSBANK	104	1721.58	cpd:C00114	Choline
KEGG/MASSBANK	184.07	251.99	cpd:C00588	Choline phosphate
KEGG/MASSBANK	226.05	95.58	cpd:C00251	Chorismate
KEGG/MASSBANK	449.1	733.72	cpd:C08604	Chrysanthemin
KEGG/MASSBANK	254.19	1053.95	cpd:C17610	Chrysanthetriol
KEGG/MASSBANK	416.11	90.19	cpd:C10316	Chrysophanol 8-O-beta-D-glucoside
KEGG/MASSBANK	358.16	602.21	cpd:C01675	Cilastatin
KEGG/MASSBANK	452.1	668.29	cpd:C10217	Cinchonain 1a
KEGG/MASSBANK	430.21	705.88	cpd:C10762	Cinegalline 2-Dihydronaph 2-diol
KEGG/MASSBANK	148.05	1131.65	cpd:C10438	Cinnamic acid 11b-Hexahydro-9-methoxy-2H-benzo[a]quinolizine-3-carboxylic acid
KEGG/MASSBANK	385.12	89.67	cpd:C13993	Ciprofloxacin hydrochloride
KEGG/MASSBANK	162.07	1240.29	cpd:C04314	cis-1 3-Dihydrodiol 1 1-Trichloro-2 2-bis(4'-chlorophenyl)ethane
KEGG/MASSBANK	261.14	1250.01	cpd:C15462	cis-1
KEGG/MASSBANK	212.18	735.14	cpd:C18206	cis-11-Methyl-2-dodecenoic acid
KEGG/MASSBANK	385.92	1742.15	cpd:C06649	cis-2
KEGG/MASSBANK	304.06	700.89	cpd:C12316	cis-Dihydroquercetin
KEGG/MASSBANK	747.5	1737.15	cpd:C06912	Clarithromycin
KEGG/MASSBANK	198	1789.88	cpd:C06660	Clavaminic acid
KEGG/MASSBANK	446.2	349.06	cpd:C13810	Clavulone I
KEGG/MASSBANK	504.1	110.11	cpd:C11728	Clindamycin ph 8-dideoxy-beta-D-manno-octulosonate
KEGG/MASSBANK	472.12	773.16	cpd:C06915	Clofazimine
KEGG/MASSBANK	292.1	116.11	cpd:C19070	Clofop
KEGG/MASSBANK	542.13	105.6	cpd:C21334	CMP-8-amino-3
KEGG/MASSBANK	865.52	1372.03	cpd:C15761	Concanamycin A
KEGG/MASSBANK	540.31	969.59	cpd:C17766	Condurangogenin C
KEGG/MASSBANK	712.33	1079.99	cpd:C19986	Convalloside

KEGG/MASSBANK	442.17	655.37	cpd:C02822	Cortisol 21-sulfate
KEGG/MASSBANK	192.08	553.28	cpd:C20465	Coumaryl acetate
KEGG/MASSBANK	291.08	351.95	cpd:C18972	Crufomate
KEGG/MASSBANK	480.11	629.63	cpd:C17880	Cryptocyanin
KEGG/MASSBANK	261.21	1287.22	cpd:C10139	Cryptophorine
KEGG/MASSBANK	518.32	1142.11	cpd:C08798	Cucurbitacin F
KEGG/MASSBANK	120.1	1296.57	cpd:C14396	Cumene 7-di-O-beta-D-glucoside
KEGG/MASSBANK	310.17	1304.88	cpd:C06530	Cupreine
KEGG/MASSBANK	611.14	647.4	cpd:C16369	Cyanidin 3-(6-p-caffeoyl)glucoside
KEGG/MASSBANK	611.16	673.83	cpd:C20469	Cyanidin 3
KEGG/MASSBANK	595.15	636.38	cpd:C12095	Cyanidin 3-O-(6-O-p-coumaroyl)glucoside
KEGG/MASSBANK	581.15	312.77	cpd:C20468	Cyanidin 3-O-(beta-D-xylosyl-(1->2)-beta-D-galactoside)
KEGG/MASSBANK	294.08	124.74	cpd:C18974	Cyanthoate
KEGG/MASSBANK	266.18	1282.73	cpd:C06930	Cyclizine
KEGG/MASSBANK	185.06	119.85	cpd:C19117	Cyometrinil
KEGG/MASSBANK	166.1	880.26	cpd:C14147	Cyromazine
KEGG/MASSBANK	479.27	757.86	cpd:C19954	Cytochalasin B
KEGG/MASSBANK	178.14	933.47	cpd:C01487	D-Allose
KEGG/MASSBANK	580.26	634.58	cpd:C09418	Daphnoline
KEGG/MASSBANK	624.3	1427.03	cpd:C09419	Dauricine
KEGG/MASSBANK	307.06	77.64	cpd:C00239	dCMP
KEGG/MASSBANK	523.2	664.7	cpd:C07307	Deacetylipecoside
KEGG/MASSBANK	368.08	108.09	cpd:C20022	Decarbamoylgonyautoxin 1
KEGG/MASSBANK	478.28	1171.99	cpd:C07996	Dehydroemetine
KEGG/MASSBANK	552.18	93.55	cpd:C08087	Delavirdine mesylate
KEGG/MASSBANK	479.29	1238.72	cpd:C08675	Delcorine
KEGG/MASSBANK	453.27	1210.47	cpd:C08676	Delcosine
KEGG/MASSBANK	597.15	631.22	cpd:C20491	Delphinidin 3-O-beta-D-sambubioside
KEGG/MASSBANK	465.1	689.6	cpd:C12138	Delphinidin 3-O-glucoside
KEGG/MASSBANK	402.3	1012.86	cpd:C14151	delta-Tocopherol
KEGG/MASSBANK	438.35	1386.3	cpd:C21084	Demethylphylloquinol
KEGG/MASSBANK	97.1	1666.13	cpd:C02026	Deoxycytosine
KEGG/MASSBANK	252.1	105.95	cpd:C05512	Deoxyinosine
KEGG/MASSBANK	496.18	611.12	cpd:C16725	Deoxypumiloside
KEGG/MASSBANK	134.1	746.03	cpd:C01801	Deoxyribose
KEGG/MASSBANK	578.26	1166.28	cpd:C06541	Deserpidine
KEGG/MASSBANK	329.09	90.37	cpd:C01069	Desulfoglucotropeolin
KEGG/MASSBANK	598.16	108.88	cpd:C12961	Dexamethazone metasulfobenzoate sodium
KEGG/MASSBANK	179.97	82.17	cpd:C00095	D-Fructose
KEGG/MASSBANK	181.05	87.6	cpd:C00124	D-Galactose
KEGG/MASSBANK	210.04	89.26	cpd:C00818	D-Glucarate
KEGG/MASSBANK	180.1	984.68	cpd:C00031	D-Glucose
KEGG/MASSBANK	86.04	913.6	cpd:C00741	Diacetyl
KEGG/MASSBANK	361.18	1149.74	cpd:C12886	Dialicor
KEGG/MASSBANK	229.99	93.6	cpd:C06949	Diazoxide
KEGG/MASSBANK	184.03	236.65	cpd:C20125	Dibenzothiophene
KEGG/MASSBANK	298.12	1020.48	cpd:C07332	Dibenzylsuccinate
KEGG/MASSBANK	268.01	1720.83	cpd:C14292	Dichlorophen
KEGG/MASSBANK	303.91	1742.42	cpd:C07459	Dichlorphenamide
KEGG/MASSBANK	327.09	94.51	cpd:C11235	Diclobutrazol

KEGG/MASSBANK	340.03	114.77	cpd:C11021	Diclofop methyl	
KEGG/MASSBANK	412.02	111.52	cpd:C01344	dIDP	
KEGG/MASSBANK	106.06	693.35	cpd:C14689	Diethylene glycol	
KEGG/MASSBANK	444.17	108.37	cpd:C16807	Difenacoum	
KEGG/MASSBANK	796.4	1184.52	cpd:C08861	Diginatin	
KEGG/MASSBANK	764	77.8	cpd:C06955	Digitoxin	
KEGG/MASSBANK	208.06	628.23	cpd:C02147	Dihydrolipoate	
KEGG/MASSBANK	314.11	87.86	cpd:C00921	Dihydropteroate	
KEGG/MASSBANK	338.11	114.89	cpd:C18783	Dimefuron	
KEGG/MASSBANK	541.2	674.64	cpd:C13441	Dimethylaminoethyl reserpilinate dihydrochloride	
KEGG/MASSBANK	146.08	690.65	cpd:C03114	Dimethylbenzimidazole	
KEGG/MASSBANK	121.03	674.75	cpd:C03392	Dimethylsulfonioacetate	
KEGG/MASSBANK	374.08	104.76	cpd:C10332	Diospyrin	
KEGG/MASSBANK	452.25	632.84	cpd:C07872	Diphenoxylate	
KEGG/MASSBANK	274.03	90.58	cpd:C18400	Disulfoton	
KEGG/MASSBANK	154.01	1782.63	cpd:C00950	Dithioerythritol	
KEGG/MASSBANK	150.04	89.7	cpd:C00476	D-Lyxose	
KEGG/MASSBANK	566.28	674.03	cpd:C15662	Dmp 323	
KEGG/MASSBANK	318.06	108.95	cpd:C07488	DMPP	
KEGG/MASSBANK	340.3	1347.54	cpd:C08281	Docosanoic acid	
KEGG/MASSBANK	199.19	1188.52	cpd:C13831	Dodecanamide	
KEGG/MASSBANK	153.08	107.43	cpd:C03758	Dopamine	
KEGG/MASSBANK	195	1785.43	cpd:C00822	Dopaquinone	
KEGG/MASSBANK	279.16	1270.62	cpd:C06971	Doxepin	6-dideoxy-L-erythro-hexos-3-ulose
KEGG/MASSBANK	444.15	648.09	cpd:C06973	Doxycycline	
KEGG/MASSBANK	150.02	148.45	cpd:C00121	D-Ribose	
KEGG/MASSBANK	530.07	679.93	cpd:C11932	dTDP-2	
KEGG/MASSBANK	532.09	668.28	cpd:C18636	dTDP-2-deoxy-beta-L-fucose	
KEGG/MASSBANK	275.12	814.37	cpd:C10662	Dubinidine	
KEGG/MASSBANK	150.05	88.33	cpd:C00181	D-Xylose	
KEGG/MASSBANK	475.14	86.74	cpd:C11595	E3040 glucuronide	
KEGG/MASSBANK	550.42	1590.26	cpd:C08592	Echinenone	
KEGG/MASSBANK	231.05	88.93	cpd:C19102	Eglinazine	
KEGG/MASSBANK	476.27	1120.61	cpd:C09420	Emetamine	
KEGG/MASSBANK	578.14	280.61	cpd:C10221	Epicatechin-(4beta->8)-ent-epicatechin	
KEGG/MASSBANK	329.07	208.03	cpd:C11229	Epoxiconazole	
KEGG/MASSBANK	608.26	1359.24	cpd:C21192	Epoxypheophorbide a	
KEGG/MASSBANK	585.22	844.87	cpd:C18640	epsilon-Rhodomycin T	
KEGG/MASSBANK	230.1	138.43	cpd:C05570	Ergothioneine	
KEGG/MASSBANK	419.1	136.08	cpd:C18147	Esfenvalerate	
KEGG/MASSBANK	396.27	1539.62	cpd:C14640	Estradiol 17beta-cyclopentylpropionate	
KEGG/MASSBANK	144.03	90.87	cpd:C07833	Ethchlorvynol	
KEGG/MASSBANK	286.09	1278	cpd:C18829	Ethofumesate	
KEGG/MASSBANK	398.09	102.97	cpd:C18440	Ethoxysulfuron	
KEGG/MASSBANK	215.15	893.43	cpd:C18830	Ethyl 3-(N-butylacetamido)propionate	
KEGG/MASSBANK	292.06	85.22	cpd:C18985	Etrimfos	
KEGG/MASSBANK	245.98	98.19	cpd:C17169	Eudistomin N	
KEGG/MASSBANK	412.13	610.59	cpd:C09416	Eupachlorin	
KEGG/MASSBANK	450.09	111.44	cpd:C10593	Exserohilone	
KEGG/MASSBANK	374.13	85.77	cpd:C18591	Famoxadone	

KEGG/MASSBANK	723.46	1402.89	cpd:C19957	Fasciculol E
KEGG/MASSBANK	253.2	1061.41	cpd:C11207	Fenapanil
KEGG/MASSBANK	360.11	89.32	cpd:C07586	Fenofibrate
KEGG/MASSBANK	522.14	837.22	cpd:C02539	Fenoprofen calcium
KEGG/MASSBANK	361.1	109.42	cpd:C11024	Fenoxaprop-ethyl
KEGG/MASSBANK	278.02	86.53	cpd:C14420	Fenthion
KEGG/MASSBANK	310.06	627.94	cpd:C17135	Filiforminol
KEGG/MASSBANK	435.94	79.44	cpd:C11099	Fipronil
KEGG/MASSBANK	224	79.37	cpd:C00766	Flavanone
KEGG/MASSBANK	206.02	106.1	cpd:C18012	Flaviolin
KEGG/MASSBANK	238	106.77	cpd:C01495	Flavonol
KEGG/MASSBANK	562.17	701.27	cpd:C15581	Flavonol 3-O-beta-D-glucosyl-(1->2)-beta-D-glucoside
KEGG/MASSBANK	724.22	686.73	cpd:C15582	Flavonol 3-O-beta-D-glucosyl-(1->2)-beta-D-glucosyl-(1->2)-beta-D-glucoside
KEGG/MASSBANK	451.16	115.59	cpd:C14524	Flucythrinate
KEGG/MASSBANK	258.11	120.65	cpd:C19031	Fluenetil
KEGG/MASSBANK	488.04	74.67	cpd:C18430	Flufenoxuron
KEGG/MASSBANK	494.2	677.49	cpd:C07007	Fluocinonide
KEGG/MASSBANK	166.08	118.8	cpd:C07715	Fluorene
KEGG/MASSBANK	502.1	634.72	cpd:C10989	Fluvalinate
KEGG/MASSBANK	82.05	1327.02	cpd:C07837	Fomepizole
KEGG/MASSBANK	776.28	759.13	cpd:C01001	Formylmethanofuran
KEGG/MASSBANK	356.09	91.32	cpd:C12451	Fridamycin E
KEGG/MASSBANK	312.22	1106.06	cpd:C09191	Fruticosonine
KEGG/MASSBANK	116	97.73	cpd:C00122	Fumarate
KEGG/MASSBANK	479.24	853.13	cpd:C20630	Fumitremorgin B
KEGG/MASSBANK	288.05	647.8	cpd:C14304	Furamizole
KEGG/MASSBANK	225.04	88.52	cpd:C07999	Furazolidone
KEGG/MASSBANK	170	107.32	cpd:C01424	Gallate
KEGG/MASSBANK	562.15	741.03	cpd:C17775	Gambiriin B1
KEGG/MASSBANK	684	77.35	cpd:C18005	gamma-L-Gluta 6-dideoxy-alpha-D-mannose
KEGG/MASSBANK	250.06	110.71	cpd:C00669	gamma-L-Glutamyl-L-cysteine
KEGG/MASSBANK	396.16	716.23	cpd:C10063	Gartanin
KEGG/MASSBANK	630.11	721.1	cpd:C20672	GDP-4-acetamido-4
KEGG/MASSBANK	601.1	667.57	cpd:C21368	GDP-valienol
KEGG/MASSBANK	263.07	83.55	cpd:C07650	Gemcitabine
KEGG/MASSBANK	388.14	796.23	cpd:C09781	Geniposide
KEGG/MASSBANK	598.19	700.48	cpd:C11639	Geniposide pentaacetate
KEGG/MASSBANK	566.12	82.19	cpd:C10048	Ginkgetin
KEGG/MASSBANK	440.1	715.21	cpd:C07603	Ginkgolide C
KEGG/MASSBANK	638.44	1433.97	cpd:C20780	Ginsenoside F1
KEGG/MASSBANK	347.03	75.91	cpd:C08413	Glucolepidiin
KEGG/MASSBANK	610.18	664.39	cpd:C19690	Glutathione amide disulfide
KEGG/MASSBANK	424.37	1633.72	cpd:C16967	Glutinone
KEGG/MASSBANK	338.28	1327.99	cpd:C19426	Glycidyl oleate
KEGG/MASSBANK	76	402.6	cpd:C00160	Glycolate
KEGG/MASSBANK	518.19	751.17	cpd:C07667	Gossypol
KEGG/MASSBANK	444.11	163.07	cpd:C06799	Granaticin
KEGG/MASSBANK	417.1	680.71	cpd:C20799	Grixazone B
KEGG/MASSBANK	213.15	1130.07	cpd:C07035	Guanadrel
KEGG/MASSBANK	244.12	742.99	cpd:C06657	Guanidinoproclavaminic acid

KEGG/MASSBANK	283.09	111.8	cpd:C00387	Guanosine
KEGG/MASSBANK	510.2	649.13	cpd:C15623	GW 409544
KEGG/MASSBANK	502.32	942.48	cpd:C15622	GW 7647
KEGG/MASSBANK	652.29	1540.59	cpd:C09208	Haplophytine
KEGG/MASSBANK	214.11	740.94	cpd:C06536	Harmaline
KEGG/MASSBANK	574.29	935.88	cpd:C11225	Herbimycin
KEGG/MASSBANK	252.16	1093.14	cpd:C10926	Hexazinone
KEGG/MASSBANK	101.12	1720.13	cpd:C08306	Hexylamine
KEGG/MASSBANK	440.18	251.84	cpd:C21127	Holyrine A
KEGG/MASSBANK	569.13	610.79	cpd:C15343	HR1917
KEGG/MASSBANK	204.19	1293.03	cpd:C09684	Humulene
KEGG/MASSBANK	292.07	309.93	cpd:C14204	Hydroxyflutamide
KEGG/MASSBANK	130.05	170.27	cpd:C01157	Hydroxyproline
KEGG/MASSBANK	374.18	677.05	cpd:C07045	Hydroxyzine
KEGG/MASSBANK	536.39	1034.89	cpd:C07608	Hyperforin
KEGG/MASSBANK	109.02	127.15	cpd:C00519	Hypotaurine
KEGG/MASSBANK	151.04	88.7	cpd:C02419	Hypotaurocyamine
KEGG/MASSBANK	454.17	649.43	cpd:C13426	I-123 BMIPP
KEGG/MASSBANK	356.06	109.02	cpd:C13703	IAA-94
KEGG/MASSBANK	308.27	1238.19	cpd:C16525	Icosadienoic acid
KEGG/MASSBANK	310.29	1389.47	cpd:C16526	Icosenoic acid
KEGG/MASSBANK	449.22	597.89	cpd:C17485	Ignavine
KEGG/MASSBANK	469.16	662.62	cpd:C04611	Indol-3-ylacetyl-myo-inositol L-arabinoside
KEGG/MASSBANK	312.07	676.63	cpd:C03280	Inosine-5'-carboxylate
KEGG/MASSBANK	526.17	753.12	cpd:C09114	Inumakilactone A glycoside
KEGG/MASSBANK	506.3	1301.49	cpd:C20009	Iriomoteolide 1a
KEGG/MASSBANK	522.2	658.23	cpd:C08769	Isobrucein A
KEGG/MASSBANK	392.22	1402.11	cpd:C09115	Isodomedin
KEGG/MASSBANK	68.06	1669.77	cpd:C16521	Isoprene
KEGG/MASSBANK	306.06	77.66	cpd:C11112	Isoprothiolane sulfoxide
KEGG/MASSBANK	528.38	1415.72	cpd:C15943	Isorenieratene
KEGG/MASSBANK	422.19	726.46	cpd:C16809	Isovaltrate
KEGG/MASSBANK	416.27	1387.3	cpd:C17989	Istamycin A3
KEGG/MASSBANK	566.15	649.52	cpd:C18212	Jamaicamide A
KEGG/MASSBANK	164.12	901.03	cpd:C08490	Jasmone
KEGG/MASSBANK	565.4	1382.18	cpd:C13931	JSTX-3
KEGG/MASSBANK	364.09	87.48	cpd:C10636	Justicidin B
KEGG/MASSBANK	286.05	829.97	cpd:C05903	Kaempferol
KEGG/MASSBANK	610.15	674.69	cpd:C12634	Kaempferol 3-O-beta-D-glucosyl-(1->2)-beta-D-glucoside
KEGG/MASSBANK	740.17	703.97	cpd:C10233	Kandelin A-1
KEGG/MASSBANK	530.3	986.09	cpd:C17615	Kukoamine A
KEGG/MASSBANK	490.1	706.84	cpd:C05560	L-2-Aminoadipate adenylate
KEGG/MASSBANK	560.23	662.7	cpd:C08870	Labriformidin
KEGG/MASSBANK	89	1790.51	cpd:C00041	L-Alanine
KEGG/MASSBANK	406.09	1016.58	cpd:C10075	Lancerin
KEGG/MASSBANK	832.5	1735.87	cpd:C15679	Lankamycin
KEGG/MASSBANK	554.2	826.1	cpd:C17686	Lappaol C
KEGG/MASSBANK	174.11	89.54	cpd:C00062	L-Arginine
KEGG/MASSBANK	227.08	88.74	cpd:C00826	L-Arogenate
KEGG/MASSBANK	132.1	107.54	cpd:C00152	L-Asparagine

KEGG/MASSBANK	133.04	89.62	cpd:C00049	L-Aspartate
KEGG/MASSBANK	625.3	1425.41	cpd:C02166	Leukotriene C4
KEGG/MASSBANK	236.08	107.25	cpd:C02700	L-Formylkynurenine
KEGG/MASSBANK	146.07	89.66	cpd:C00064	L-Glutamine
KEGG/MASSBANK	196	1793.54	cpd:C00800	L-Gulonate
KEGG/MASSBANK	268.1	108.38	cpd:C01817	L-Homocystine
KEGG/MASSBANK	506.2	753.61	cpd:C01593	Limonoate
KEGG/MASSBANK	280.24	1242.01	cpd:C01595	Linoleate
KEGG/MASSBANK	131.1	107.15	cpd:C00407	L-Isoleucine
KEGG/MASSBANK	208.08	429.38	cpd:C00328	L-Kynurenine
KEGG/MASSBANK	132.03	1780.46	cpd:C00123	L-Leucine
KEGG/MASSBANK	146.11	1228.01	cpd:C00047	L-Lysine
KEGG/MASSBANK	149.05	87.76	cpd:C00073	L-Methionine
KEGG/MASSBANK	390.2	1014.06	cpd:C01433	Loganin
KEGG/MASSBANK	422.16	345.71	cpd:C07072	Losartan
KEGG/MASSBANK	314.18	1173.45	cpd:C17567	Lotusine
KEGG/MASSBANK	165.08	118.42	cpd:C00079	L-Phenylalanine
KEGG/MASSBANK	129.08	90.24	cpd:C00408	L-Pipecolate
KEGG/MASSBANK	115.06	89.67	cpd:C00148	L-Proline
KEGG/MASSBANK	105	90.38	cpd:C00065	L-Serine
KEGG/MASSBANK	199.02	91.31	cpd:C12147	L-Threonine O-3-phosphate
KEGG/MASSBANK	204.1	161.41	cpd:C00078	L-Tryptophan
KEGG/MASSBANK	181.07	96.23	cpd:C00082	L-Tyrosine
KEGG/MASSBANK	442.18	1149.11	cpd:C10474	Lusitanicoside
KEGG/MASSBANK	574.11	125.3	cpd:C16763	Luteoskyrin
KEGG/MASSBANK	117	92	cpd:C00183	L-Valine
KEGG/MASSBANK	166.05	117.84	cpd:C05411	L-Xylonate
KEGG/MASSBANK	247.19	1286.3	cpd:C09883	Lycopodine
KEGG/MASSBANK	291.07	315.33	cpd:C08531	Lycoricidine
KEGG/MASSBANK	93.92	84.99	cpd:C07755	Magnesium chloride
KEGG/MASSBANK	584.23	644.25	cpd:C03516	Magnesium protoporphyrin
KEGG/MASSBANK	113.01	92.32	cpd:C01384	Maleic acid
KEGG/MASSBANK	442.16	109.3	cpd:C09013	Mallotochromene
KEGG/MASSBANK	612.4	1723.38	cpd:C09127	Mancinellin
KEGG/MASSBANK	524.23	617.13	cpd:C09132	Mascaroside
KEGG/MASSBANK	390.19	996.07	cpd:C07116	Meclizine
KEGG/MASSBANK	518.14	81.16	cpd:C16224	Medicarpin 3-O-glucoside-6'-malonate
KEGG/MASSBANK	304.07	105.3	cpd:C19442	Medphalan
KEGG/MASSBANK	378.12	87.53	cpd:C07633	Mefloquine
KEGG/MASSBANK	126.07	1737.76	cpd:C08737	Melamine
KEGG/MASSBANK	522.17	89.52	cpd:C09501	Melampodinin
KEGG/MASSBANK	310.11	105.68	cpd:C09502	Mellitoxin
KEGG/MASSBANK	172.05	102.84	cpd:C05377	Menadione
KEGG/MASSBANK	322.15	576	cpd:C12755	Mequitazine
KEGG/MASSBANK	123.95	1727.63	cpd:C07295	Methanearsonous acid
KEGG/MASSBANK	95.99	88.11	cpd:C11145	Methanesulfonic acid
KEGG/MASSBANK	266.05	103.43	cpd:C08506	Methoxybrassinin
KEGG/MASSBANK	354.28	1237.64	cpd:C12280	Methyl acetyl ricinoleate
KEGG/MASSBANK	256.05	129.64	cpd:C00225	Methyl viologen
KEGG/MASSBANK	72.02	79.06	cpd:C00546	Methylglyoxal

KEGG/MASSBANK	309.19	997.96	cpd:C07915	Metipranolol
KEGG/MASSBANK	429.27	1092.41	cpd:C07652	Mifepristone
KEGG/MASSBANK	352.26	1323.84	cpd:C09137	Montanol
KEGG/MASSBANK	437.15	108.61	cpd:C08054	Moxifloxacin hydrochloride
KEGG/MASSBANK	580.17	99.35	cpd:C08928	Mulberrofuran C
KEGG/MASSBANK	420.08	106.88	cpd:C08560	Musca-aurin-II
KEGG/MASSBANK	517	75.65	cpd:C17009	Mycalamide B
KEGG/MASSBANK	727.41	1121.93	cpd:C15680	Mycinamicin II
KEGG/MASSBANK	695.42	969.35	cpd:C18846	Mycinamicin IV
KEGG/MASSBANK	756.2	603.27	cpd:C10479	Myricoside
KEGG/MASSBANK	415.3	1434.74	cpd:C12158	Myxalamid A
KEGG/MASSBANK	403.2	1029.75	cpd:C12221	Myxochelin B
KEGG/MASSBANK	487.2	670.58	cpd:C15674	Myxothiazol A
KEGG/MASSBANK	274.13	849.11	cpd:C03296	N2-Succinyl-L-arginine
KEGG/MASSBANK	188.12	391.28	cpd:C02727	N6-Acetyl-L-lysine
KEGG/MASSBANK	481.25	707.84	cpd:C11361	N-Acetylleukotriene E4
KEGG/MASSBANK	494.13	659.77	cpd:C03709	N-Adenylyl-L-phenylalanine
KEGG/MASSBANK	128.06	88	cpd:C00829	Naphthalene
KEGG/MASSBANK	307.07	123.75	cpd:C08533	Narciclasine
KEGG/MASSBANK	278.14	1074.16	cpd:C03001	N-Benzoyl-D-arginine
KEGG/MASSBANK	620.25	1445.68	cpd:C20138	N-Demethylansamitocin P-3
KEGG/MASSBANK	303.02	76.39	cpd:C12862	Nedaplatin
KEGG/MASSBANK	414.17	716.14	cpd:C10707	Neoisostegane
KEGG/MASSBANK	266.12	677.88	cpd:C07263	Nevirapine
KEGG/MASSBANK	251.08	462.3	cpd:C02564	N-Feruloylglycine
KEGG/MASSBANK	143.02	1753.22	cpd:C18232	N-Formylmaleamic acid
KEGG/MASSBANK	334.06	87.32	cpd:C00455	Nicotinamide D-ribonucleotide
KEGG/MASSBANK	450.23	607.69	cpd:C17030	Nigakilactone E
KEGG/MASSBANK	418.17	381.08	cpd:C07267	Nimodipine
KEGG/MASSBANK	312.18	1007.38	cpd:C11810	Norajmaline
KEGG/MASSBANK	511.19	671.56	cpd:C19139	Norbormide
KEGG/MASSBANK	173.12	1126.81	cpd:C15476	Norselegiline
KEGG/MASSBANK	612.23	615.73	cpd:C05080	Novobiocin
KEGG/MASSBANK	231.01	115.41	cpd:C03624	N-Phosphohypotaurocyamine
KEGG/MASSBANK	362.05	111.54	cpd:C13833	NS 1619
KEGG/MASSBANK	390.07	111.25	cpd:C20684	O-Carbamoyladenylate
KEGG/MASSBANK	283.29	1540.92	cpd:C13846	Octadecanamide
KEGG/MASSBANK	144.1	982.17	cpd:C06423	Octanoic acid
KEGG/MASSBANK	129.15	1722.81	cpd:C01740	Octylamine
KEGG/MASSBANK	804.5	1408.43	cpd:C01945	Okadaic acid
KEGG/MASSBANK	632.39	1351.91	cpd:C08964	Oleanoic acid 3-O-glucuronide
KEGG/MASSBANK	790.52	1537.23	cpd:C11311	Oligomycin A
KEGG/MASSBANK	368.03	112.85	cpd:C01103	Orotidine 5'-phosphate
KEGG/MASSBANK	97.98	102.71	cpd:C00009	Orthophosphate
KEGG/MASSBANK	204.03	77.41	cpd:C05533	Oxaloglutarate
KEGG/MASSBANK	496.12	655.31	cpd:C13055	Oxytetracycline hydrochloride
KEGG/MASSBANK	439.22	728.01	cpd:C10357	Parsonsine
KEGG/MASSBANK	134.04	88.47	cpd:C06575	p-Cymene
KEGG/MASSBANK	188.09	710.9	cpd:C10733	Peganine
KEGG/MASSBANK	579.15	284.04	cpd:C16368	Pelargonidin 3-(6-p-coumaroyl)glucoside

KEGG/MASSBANK	579.17	686.13	cpd:C12644	Pelargonidin 3-O-rutinoside	
KEGG/MASSBANK	599.32	1124.3	cpd:C20597	Penitrem E	5-dicarboxylate
KEGG/MASSBANK	248.11	682.04	cpd:C07423	Pentobarbital s	6-dicarboxylic acid
KEGG/MASSBANK	479.1	687.96	cpd:C12139	Petunidin 3-O-glucoside	
KEGG/MASSBANK	266.06	1127.25	cpd:C18252	Phenanthrene-4	
KEGG/MASSBANK	268.05	1720.21	cpd:C12119	Phenazine-1	
KEGG/MASSBANK	121.09	169.47	cpd:C05332	Phenethylamine	
KEGG/MASSBANK	94.04	77.67	cpd:C00146	Phenol	
KEGG/MASSBANK	174.01	100.12	cpd:C02734	Phenolic phosphate	
KEGG/MASSBANK	167.04	87.6	cpd:C03719	Phenylacetothiohydroximate	
KEGG/MASSBANK	122.05	108.02	cpd:C16200	Phenylboronic acid	
KEGG/MASSBANK	206.1	1014.04	cpd:C07499	Phenylethylmalonamide	
KEGG/MASSBANK	592.27	1460.26	cpd:C18021	Pheophorbide a	
KEGG/MASSBANK	870.57	1676.14	cpd:C05797	Pheophytin a	
KEGG/MASSBANK	211.04	145.89	cpd:C02305	Phosphocreatine	
KEGG/MASSBANK	96.99	87.16	cpd:C02306	Phosphoramidate	
KEGG/MASSBANK	254.09	95.32	cpd:C16584	p-Hydroxyfelbamate	
KEGG/MASSBANK	183.04	209.72	cpd:C17239	p-Hydroxyphenylacetothiohydroximate	
KEGG/MASSBANK	450.35	1564.34	cpd:C02059	Phylloquinone	
KEGG/MASSBANK	317.29	1104.18	cpd:C12144	Phytosphingosine	
KEGG/MASSBANK	376.27	1355.34	cpd:C20764	Phytyl phosphate	
KEGG/MASSBANK	414.13	105.98	cpd:C10871	Picropodophyllin	
KEGG/MASSBANK	248.15	1728.72	cpd:C07445	Pindolol	N-(Dimethylamino)phenyldiazonium fluoroborate
KEGG/MASSBANK	212.08	85.44	cpd:C01745	Pinosylvin	
KEGG/MASSBANK	83.07	122.82	cpd:C06181	Piperideine	
KEGG/MASSBANK	235.09	105.85	cpd:C13681	p-N	
KEGG/MASSBANK	374.11	129.12	cpd:C08565	Portulacaxanthin II	
KEGG/MASSBANK	578.36	1305.43	cpd:C13554	Prednisolone 21-all-cis-farnesylate	
KEGG/MASSBANK	576.13	749.09	cpd:C10237	Proanthocyanidin A2	
KEGG/MASSBANK	235.17	1214.69	cpd:C07401	Procainamide	
KEGG/MASSBANK	257.13	847	cpd:C07376	Procarbazine hydrochloride	
KEGG/MASSBANK	347.11	113.85	cpd:C19065	Profluralin	
KEGG/MASSBANK	229.15	107.32	cpd:C11707	Pronethalol	
KEGG/MASSBANK	60	1790.12	cpd:C01845	Propan-2-ol	
KEGG/MASSBANK	58.04	8.92	cpd:C00479	Propanal	
KEGG/MASSBANK	60.06	58.26	cpd:C05979	Propane-1-ol	
KEGG/MASSBANK	443.12	646.8	cpd:C18886	Propaquizafop	
KEGG/MASSBANK	476.39	1630.6	cpd:C20716	Protopanaxatriol	
KEGG/MASSBANK	428.18	701.52	cpd:C08951	Pseudorhodomyrtoxin	
KEGG/MASSBANK	862.5	1410.29	cpd:C09233	Psychotridine	
KEGG/MASSBANK	594.18	671.99	cpd:C07389	Pyrantel pamoate	
KEGG/MASSBANK	169	117.83	cpd:C00314	Pyridoxine	
KEGG/MASSBANK	221.09	105.77	cpd:C02587	Pyrimidodiazepine	
KEGG/MASSBANK	88	1781.24	cpd:C00022	Pyruvate	
KEGG/MASSBANK	302	79.58	cpd:C00389	Quercetin	
KEGG/MASSBANK	364.24	1154.63	cpd:C07619	Quinestrol	
KEGG/MASSBANK	372.09	407.64	cpd:C18530	Quizalofop-ethyl	
KEGG/MASSBANK	228.03	99.81	cpd:C16457	Radium-228	
KEGG/MASSBANK	626.27	1168.57	cpd:C18022	Red chlorophyll catabolite	
KEGG/MASSBANK	388.21	633.96	cpd:C17452	Rehmaionoside C	

KEGG/MASSBANK	229.04	94.38	cpd:C11178	Resazurin
KEGG/MASSBANK	608.27	1440.12	cpd:C06539	Reserpine
KEGG/MASSBANK	228.08	1142.61	cpd:C03582	Resveratrol 5'-phosphate
KEGG/MASSBANK	316.06	712.21	cpd:C10176	Rhamnetin
KEGG/MASSBANK	376.14	650.73	cpd:C00255	Riboflavin
KEGG/MASSBANK	438.1	632.55	cpd:C16071	Riboflavin cyclic-4'
KEGG/MASSBANK	822.41	1155.2	cpd:C06688	Rifampicin
KEGG/MASSBANK	516.16	100.66	cpd:C18235	S-(Hydroxymethyl)mycothiol
KEGG/MASSBANK	400.11	104.08	cpd:C03725	S-Acetylphosphopantetheine
KEGG/MASSBANK	122.04	353.96	cpd:C06202	Salicylaldehyde
KEGG/MASSBANK	138.03	353.95	cpd:C00805	Salicylate
KEGG/MASSBANK	436.15	108.48	cpd:C17869	Sanggenon A
KEGG/MASSBANK	394.17	604.81	cpd:C12228	Sanguilutine
KEGG/MASSBANK	402.12	95.61	cpd:C12311	Saphenamycin
KEGG/MASSBANK	594.16	710.5	cpd:C08064	Saponarin
KEGG/MASSBANK	504.16	86.9	cpd:C08782	Sergeolide
KEGG/MASSBANK	279.18	1149.93	cpd:C09901	Serratine
KEGG/MASSBANK	426.09	653.26	cpd:C05526	S-Glutathionyl-L-cysteine
KEGG/MASSBANK	215.99	98.55	cpd:C04399	S-Methyl-3-phospho-1-thio-D-glycerate
KEGG/MASSBANK	106.01	408.83	cpd:C03173	S-Methylthioglycolate
KEGG/MASSBANK	495.15	109.83	cpd:C17731	Sodium folinate
KEGG/MASSBANK	160.01	79.59	cpd:C07587	Sodium salicylate
KEGG/MASSBANK	218	1794.66	cpd:C09737	Solavetivone
KEGG/MASSBANK	458.38	1360.63	cpd:C08980	Soyasapogenol B
KEGG/MASSBANK	494.4	1618.25	cpd:C20475	Sporulenol
KEGG/MASSBANK	627.46	1537.4	cpd:C16841	Squalamine
KEGG/MASSBANK	267.08	105.75	cpd:C03539	S-Ribosyl-L-homocysteine
KEGG/MASSBANK	436.26	695.43	cpd:C03805	Stearoylglycerone phosphate
KEGG/MASSBANK	252.2	1354.34	cpd:C17502	Sugetriol
KEGG/MASSBANK	128.1	126.42	cpd:C07288	Sulcatol
KEGG/MASSBANK	332.06	103.08	cpd:C18386	Sulfadimethoxine sodium
KEGG/MASSBANK	280.06	100.78	cpd:C12616	Sulfamethopyrazine
KEGG/MASSBANK	260.05	91.81	cpd:C07320	Suprofen
KEGG/MASSBANK	481.13	109	cpd:C11751	Talampicillin
KEGG/MASSBANK	448.36	1634.01	cpd:C15791	Teasterone
KEGG/MASSBANK	582.1	81.64	cpd:C12040	Telomestatin
KEGG/MASSBANK	288.04	110.24	cpd:C18693	Terbufos
KEGG/MASSBANK	246	81.24	cpd:C18240	Tetrachlorocatechol
KEGG/MASSBANK	228.2	1404.34	cpd:C06424	Tetradecanoic acid
KEGG/MASSBANK	188.03	94.86	cpd:C20385	Tetranorbiotin
KEGG/MASSBANK	243.95	1725.9	cpd:C18945	Thicyofen
KEGG/MASSBANK	142.01	76.75	cpd:C02595	Thien-2-ylacetate
KEGG/MASSBANK	137.03	87.74	cpd:C16281	Thiobenzamide
KEGG/MASSBANK	248.04	85.32	cpd:C19036	Thionazin
KEGG/MASSBANK	342.05	103.73	cpd:C14432	Thiophanate-methyl
KEGG/MASSBANK	253.08	143.31	cpd:C01619	Thiorphan
KEGG/MASSBANK	189.05	94.01	cpd:C07641	Thiotepa
KEGG/MASSBANK	242.09	83.9	cpd:C00214	Thymidine
KEGG/MASSBANK	375.13	84.98	cpd:C07503	Tiagabine
KEGG/MASSBANK	263.05	91.89	cpd:C07140	Ticlopidine

KEGG/MASSBANK	79.94	1723.95	cpd:C13409	Titanium dioxide
KEGG/MASSBANK	562.23	805.37	cpd:C15659	TMC 126
KEGG/MASSBANK	357.06	109.15	cpd:C01621	Tolrestat
KEGG/MASSBANK	172.02	91.14	cpd:C06677	Toluene-4-sulfonate
KEGG/MASSBANK	849.52	1529.98	cpd:C16904	Tolytoxin
KEGG/MASSBANK	193.06	214.39	cpd:C16789	Toxoflavine
KEGG/MASSBANK	228.1	111.57	cpd:C16308	Traumatic acid
KEGG/MASSBANK	528.16	649.54	cpd:C09973	Tremulacin
KEGG/MASSBANK	294.04	109.97	cpd:C10406	Tricrozarin A
KEGG/MASSBANK	189.04	175.37	cpd:C18492	Tricyclazole
KEGG/MASSBANK	318.28	1351.59	cpd:C07861	Tridihexethyl
KEGG/MASSBANK	562.25	695.42	cpd:C09669	Trilobine
KEGG/MASSBANK	298.15	1190.38	cpd:C07172	Trimeprazine
KEGG/MASSBANK	398.24	1242.85	cpd:C14446	Tris(butoxyethyl)phosphate
KEGG/MASSBANK	141.12	1444.46	cpd:C00729	Tropine
KEGG/MASSBANK	394.27	1539.4	cpd:C12000	Tylactone
KEGG/MASSBANK	687.05	74.98	cpd:C20245	UDP-N-acetyl-alpha-D-glucosamine 3'-phosphate
KEGG/MASSBANK	162.03	137.18	cpd:C09315	Umbelliferone
KEGG/MASSBANK	393.28	1360.3	cpd:C12023	Undecylprodigiosin
KEGG/MASSBANK	112	93.52	cpd:C00106	Uracil
KEGG/MASSBANK	168.03	1777.11	cpd:C00366	Urate
KEGG/MASSBANK	450.28	1346.79	cpd:C09250	Usambarine
KEGG/MASSBANK	234.16	1214.47	cpd:C09743	Valerenic acid
KEGG/MASSBANK	168.04	90.29	cpd:C06672	Vanillate
KEGG/MASSBANK	454.28	1175.86	cpd:C07188	Verapamil
KEGG/MASSBANK	408.18	662.42	cpd:C09579	Vernoflexuoside
KEGG/MASSBANK	364.04	102.84	cpd:C00655	Xanthosine 5'-phosphate
KEGG/MASSBANK	152	90.16	cpd:C00379	Xylitol
KEGG/MASSBANK	244.19	1236.83	cpd:C07913	Xylometazoline
KEGG/MASSBANK	412.11	109.38	cpd:C07568	Ziprasidone

Tables S2. The metabolomic pathways associated with wet and dry season. The influence of the pathways and the suprafamily are indicated by the arrows. ↑ for 0.3-0.5 values in loadings of the PCA, ↑↑ for 0.5-0.8 values ↑↑↑ , for >0.8 values. ↓ for -0.3--0.5 values, ↓↓ for -0.5--0.8 values, ↓↓↓ for<-0.8 values. The categories of the suprafamily and the metabolomic pathway are based on the KEGG service.

	Seasson	DRY	WET
**Suprafamily**	**Metabolomic Pathway**		
Amino acid metabolism	Tyrosine metabolism	-	↑↑↑
Amino acid metabolism	Lysine biosynthesis	-	↑↑↑
Amino acid metabolism	Cysteine and methionine metabolism	-	↑
Biosynthesis of other secondary metabolites	Isoflavonoid biosynthesis	↓↓↓	↑↑↑
Biosynthesis of other secondary metabolites	Isoquinoline alkaloid biosynthesis	↓↓	↑↑
Biosynthesis of other secondary metabolites	Indole alkaloid biosynthesis	↓↓	↑↑
Biosynthesis of other secondary metabolites	Indole diterpene alkaloid biosynthesis	-	-
Biosynthesis of other secondary metabolites	Caffeine metabolism	-	-
Biosynthesis of other secondary metabolites	Streptomycin biosynthesis	↓	↑
Biosynthesis of other secondary metabolites	Anthocyanin biosynthesis	-	↑
Carbohydrate metabolism	Galactose metabolism	↑	-
Carbohydrate metabolism	Starch and sucrose metabolism	↑	-
Carbohydrate metabolism	Pentose phosphate pathway	↑↑	-
Carbohydrate metabolism	Propanoate metabolism	↓	↓↓↓
Carbohydrate metabolism	Glyoxylate and dicarboxylate metabolism	↑	-
Carbohydrate metabolism	Pyruvate metabolism	-	↓↓
Energy metabolism	Carbon fixation pathways in prokaryotes	↓↓↓	-
Energy metabolism	Carbon fixation in photosynthetic organisms	↑↑	-
Energy metabolism	Sulfur metabolism	-	↓↓↓
Glycan biosynthesis and metabolism	cosaminoglycan biosynthesis - heparan sulfate / hep	-	↓↓↓
Lipid metabolism	Fatty acid biosynthesis	↑↑↑	↑
Lipid metabolism	Biosynthesis of unsaturated fatty acids	↑↑	↓
Lipid metabolism	Linoleic acid metabolism	↑↑↑	-
Lipid metabolism	Steroid biosynthesis	↑↑	-
Metabolism of cofactors and vitamins	One carbon pool by folate	↓↓↓	↑
Metabolism of other amino acids	D-Alanine metabolism	↑↑	↓
Metabolism of other amino acids	Taurine and hypotaurine metabolism	-	↓↓
Metabolism of other amino acids	Selenocompound metabolism	↑↑	↓
Metabolism of other amino acids	D-Arginine and D-ornithine metabolism	-	-
Metabolism of other amino acids	Glutathione metabolism	-	-
Metabolism of terpenoids and polyketides	Biosynthesis of type II polyketide products	↓↓	↑↑↑
Metabolism of terpenoids and polyketides	Zeatin biosynthesis	-	↓↓↓
Metabolism of terpenoids and polyketides	Biosynthesis of ansamycins	↓	↑↑
Metabolism of terpenoids and polyketides	iosynthesis of 12-, 14- and 16-membered macrolide	↓	↑
Metabolism of terpenoids and polyketides	Tetracycline biosynthesis	-	↑
Metabolism of terpenoids and polyketides	Biosynthesis of vancomycin group antibiotics	-	-
Nucleotide metabolism	Pyrimidine metabolism	↓	↑
Xenobiotics biodegradation and metabolism	Ethylbenzene degradation	↓↓	↑↑
Xenobiotics biodegradation and metabolism	Toluene degradation	-	↑↑
Xenobiotics biodegradation and metabolism	Chloroalkane and chloroalkene degradation	↓↓	↑↑
Xenobiotics biodegradation and metabolism	DDT degradation	↑	↓↓
Xenobiotics biodegradation and metabolism	Dioxin degradation	↓↓	↑↑

Xenobiotics biodegradation and metabolism	Metabolism of xenobiotics by cytochrome P450	↓↓	↑↑
Xenobiotics biodegradation and metabolism	Caprolactam degradation	-	↑
Xenobiotics biodegradation and metabolism	Atrazine degradation	↑↑	-
Xenobiotics biodegradation and metabolism	Xylene degradation	-	-
Xenobiotics biodegradation and metabolism	Chlorocyclohexane and chlorobenzene degradation	-	↓
Xenobiotics biodegradation and metabolism	Nitrotoluene degradation	↓↓	-

Table S3. Species analyzed in this study

Order	Family	Genus	Species	LegSp
Laurales	Lauraceae	*Aniba*	*rosaeodora*	Aro
Apiales	Opiliaceae	*Agonandra*	*silvatica*	Asi
Fabales	Fabaceae	*Alexa*	*wachenheimii*	Awa
Rosales	Moraceae	*Brosimum*	*guianense*	Bgu
Fabales	Fabaceae	*Bocoa*	*prouacensis*	Bpr
Rosales	Moraceae	*Brosimum*	*rubescens*	Bru
Ericales	Sapotaceae	*Chrysophyllum*	*argenteum*	Car
Rosales	Chrysobalanaceae	*Couepia*	*caryophylloides*	Cca
Gentianales	Rubiaceae	*Capirona*	*decorticans*	Cde
Malvales	Malvaceae	*Catostemma*	*fragrans*	Cfr
Malpighiales	Caryocaraceae	*Caryocar*	*glabrum*	Cgl
Ericales	Sapotaceae	*Chrysophyllum*	*sanguinolentum*	Csa
Malpighiales	Euphorbiaceae	*Chaetocarpus*	*chomburgkianu*	Csc
Sapindales	Meliaceae	*Carapa*	*surispemensis*	Csu
Gentianales	Rubiaceae	*Chimarrhis*	*turbispeta*	Ctu
Fabales	Fabaceae	*Dicorynia*	*guianensis*	Dgu
Fabales	Fabaceae	*Dipteryx*	*odorata*	Dod
Malpighiales	Putranjivaceae	*Drypetes*	*variabilis*	Dva
Ericales	Lecythidaceae	*Eschweilera*	*coriacea*	Eco
Ericales	Lecythidaceae	*Eschweilera*	*decolorans*	Ede
Fabales	Fabaceae	*Eperua*	*falcata*	Efa
Fabales	Fabaceae	*Eperua*	*grandiflora*	Egr
Gentianales	Rubiaceae	*Ferdinandusa*	*paraensis*	Fpa
Malpighiales	Goupiaceae	*Goupia*	*glabra*	Ggl
Ericales	Lecythidaceae	*Gustavia*	*hexapetala*	Ghe
Rosales	Chrysobalanaceae	*Hirtella*	*bicornis*	Hbi
Fabales	Fabaceae	*hymanea*	*courbaril*	Hco
Malpighiales	Linaceae	*Hebepetalum*	*humiriifolium*	Hhu
Rosales	Moraceae	*Helicostylis*	*pedunculata*	Hpe
Fabales	Fabaceae	*Inga*	*nouraguensis*	Ino
Magnoliales	Myristicaceae	*Iryanthera*	*sagotiaspe*	Isa
Rosales	Chrysobalanaceae	*Licania*	*alba*	Lal
Rosales	Chrysobalanaceae	*Licania*	*densiflora*	Lde
Ericales	Lecythidaceae	*Lecythis*	*idatimon*	Lid
Malvales	Malvaceae	*Lueheopsis*	*rugosa*	Lru
Malpighiales	Clusiaceae	*Moronobea*	*coccinea*	Mco
Myrtales	Myrtaceae	*Myrcia*	*splendens*	Msp
Ericales	Sapotaceae	*Micropholis*	*venulosa*	Mve
Magnoliales	Annonaceae	*Oxandra*	*asbeckii*	Oas
Magnoliales	Myristicaceae	*Osteophleum*	*platyspermum*	Opl
Sapindales	Burseraceae	*Protium*	*decandrum*	Pde
Ericales	Sapotaceae	*Pouteria*	*eugeniifolia*	Peu
Fabales	Fabaceae	*Paloue*	*guianensisP*	Pgu
Sapindales	Burseraceae	*Protium*	*opacum*	Pop
Ericales	Sapotaceae	*Pradosia*	*ptychandra*	Ppt
Ericales	Sapotaceae	*Pouteria*	*retinervis*	Pre

Poales	Peraceae	*Pogonophora*	*chomburgkiaspe*	Psc
Malpighiales	Clusiaceae	*Symphonia*	*globulifera*	Sgl
Malvales	Malvaceae	*Sterculia*	*pruriens*	Spr
Laurales	Lauraceae	*Sextonia*	*rubra*	Sru
Malpighiales	Dichapetalaceae	*Tapura*	*capitulifera*	Tca
Sapindales	Sapindaceae	*Talisia*	*praealta*	Tpr
Fabales	Fabaceae	*Vouacapoua*	*americaspe*	Vam
Myrtales	Vochysiaceae	*Vochysia*	*sabatieri*	Vsa
